# Cellular effects of adamantane derivatives in P-glycoprotein leukemia cells

**DOI:** 10.1515/biol-2025-1340

**Published:** 2026-06-26

**Authors:** Natalia Dostalkova, Lucia Sofrankova, Jana Spaldova, Branislav Pavilek, Ester Pisarova, Lakatos Boris

**Affiliations:** Department of Biochemistry and Microbiology, Faculty of Chemical and Food Technology, Slovak University of Technology in Bratislava, Radlinskeho 9, SK-81237, Bratislava, Slovak Republic; Department of Organic Chemistry, Faculty of Chemical and Food Technology, Slovak University of Technology in Bratislava, Radlinskeho 9, SK-81237, Bratislava, Slovak Republic

**Keywords:** amantadine derivatives, multidrug resistance, P-glycoprotein, acute myeloid leukemia

## Abstract

Acute myeloid leukemia is a severe hematologic malignancy that predominantly affects older adults and is characterized by high biological variability. One of the main challenges in its treatment is the development of multidrug resistance, often mediated by the overexpression of P-glycoprotein (ABCB1). This transport protein actively expels cytotoxic drugs from cells, reducing the effectiveness of chemotherapy and worsening patient prognosis. Although several generations of P-glycoprotein inhibitors have been developed, their clinical use is significantly limited due to their negative side effects. In recent years, attention has shifted toward natural substances and well-known drugs with broad applications. A promising candidate is amantadine, a lipophilic compound with a stable structure, originally used to treat influenza and Parkinson’s disease. This study focuses on investigating the potential of its derivatives in combating multidrug resistance in acute myeloid leukemia. The results demonstrate that the newly synthesized adamantane derivatives, particularly E-A2, exhibit significant cytotoxic activity in acute myeloid leukemia cell lines, including P-glycoprotein-expressing resistant variants. Importantly, E-A2 was able to induce both apoptotic and necrotic cell death and maintained its activity in multidrug resistant models.

## 1 Introduction

Acute myeloid leukemia (AML) is an aggressive hematological disease characterized by the clonal expansion of immature myeloid progenitor cells in the bone marrow and peripheral blood. Although AML is a relatively rare cancer (representing approximately 1 % of all cancer cases), it is the most common hematological malignancy in adults. Most cases of this type of cancer occur in individuals older than 60 years and are characterized by biological diversity that influences the course of the disease and the response to treatment [1]. Despite advances in diagnosis and therapy, the prognosis of many patients remains unfavorable, mainly due to the frequent occurrence of relapses and the development of multidrug resistance (MDR). Table 1 provides an overview of the most common types of multidrug resistance in patients with acute myeloid leukemia, their mechanisms, and the drugs affected by MDR. MDR in acute myeloid leukemia arises due to a number of different factors, such as the influence of the tumor microenvironment and cellular mechanisms, inefficient systemic drug delivery, and upregulation of ABC (ATP-binding cassette transporters) transporters, the most important of which is P-glycoprotein (P-gp) [2]. P-gp, encoded by the *ABCB1* gene, functions as an efflux pump that actively removes cytotoxic drugs from tumor cells, thereby reducing their intracellular concentration and efficacy. In AML, increased P-gp expression has been reported in approximately one-third of patients at diagnosis and in more than half of patients in remission. Higher levels of P-gp have also been observed in certain subtypes of the disease, including secondary leukemia. In AML, P-gp expression is associated with lower complete remission rates, shorter symptom-free periods, and reduced overall survival [3]. To overcome P-glycoprotein-mediated multidrug resistance, several generations of inhibitors have been developed over the past decades to enhance the efficacy of chemotherapy in hematological malignancies. The first and second generations consisted of drugs such as verapamil, cyclosporine A, and valspodar, which proved to be functional inhibitors, but their clinical use was limited due to high toxicity and adverse pharmacokinetic interactions. The third generation of inhibitors (e.g. zosuquidar) was designed to minimize these limitations [4]. However, current research is increasingly focused on natural substances and plants secondary metabolites, such as flavonoids, terpenoids, and alkaloids – or drugs that are still commonly used in the treatment of benign diseases [4], [5], [6]. In this context, it is worth mentioning amantadine – a stable, lipophilic drug originally used against influenza and Parkinson’s disease, which, due to its wide spectrum of use, has also attracted attention in connection with oncological diseases [7], [8], [9]. In this work, we therefore decided to investigate the potential of amantadine derivatives in leukemic cell lines.

**Table 1:** Overview of the most common types of multidrug resistance. Overview of the most common types of multidrug resistance in patients with acute myeloid leukemia, their mechanism and drugs affected by MDR (adapted from Bukowski et al. [10], Niu et al. [11] and Kropp et al. [12]).

Mechanisms of drug resistance			
	Type of resistance	Mechanism of resistance	Drugs affected by resistance
Genetic mutations	Mutation in the *FLT3* gene	Persistent activation of *FLT3* leads to continuous proliferation and survival of leukemic cells	FLT3 inhibitors (midostaurin, gilteritinib)
	Mutation in the *NPM1* gene	Clonal selection of subpopulations resistant to apoptosis	Cytarabine, anthracyclines
	Mutation in the *TP53* gene	Reduced ability to activate apoptosis in response to DNA damage	Cytarabine, daunorubicin
Epigenetic changes	Hypermethylation of tumor suppressor genes	Silencing of tumor suppressor genes, reducing apoptotic response	Azacitidine, decitabine
	Histone modifications	Deacetylation or methylation affects chromatin structure and the expression of apoptosis-related genes	HDAC inhibitors, DNMT inhibitors
Changes in apoptotic pathways	Overexpression of anti-apoptotic proteins	Inhibition of mitochondrial apoptosis activation	Cytotoxic chemotherapeutics, venetoclax
	Decreased expression of pro-apoptotic proteins	Reduced ability to trigger apoptosis	Mitochondria-dependent drugs
Increased drug efflux	P-glycoprotein (P-gp, *ABCB1*)	Active transport of drugs out of the cell reduces their intracellular concentration	Doxorubicin, vinblastine, etoposide
	Breast Cancer Resistance Protein (BCRP)	Transports drugs and reduces their intracellular concentrations	Tyrosine kinase inhibitors, chemotherapeutics

## 2 Materials and methods

### 2.1 Chemicals

#### 2.1.1 Amantadine derivatives used in this study

Compounds were synthesized and provided by the Institute of Organic Chemistry, FCFT STU, Bratislava, Slovakia. LogP and error bars were calculated using the ACD/Chemsketch method. The calculation is described at https://www.acdlabs.com/products/percepta-platform/physchem-suite/logp/ (Advanced Chemistry Development, Inc. Toronto, Ontario, Canada).

##### 2.1.1.1 Chemical characterization

The chemical structures, systematic names, abbreviations, molecular weights, and calculated logP values of compounds E-A1 and E-A2 are listed in Table 2. The physicochemical characterization of the studied compounds (E-A1 and E-A2) was performed using standard analytical techniques. Melting points were determined using a Boetius apparatus and were uncorrected. Nuclear magnetic resonance (^1^H and ^13^C NMR) spectra were recorded on a Varian Unity Inova 300 MHz spectrometer, with chemical shifts referenced to tetramethylsilane (TMS) or residual solvent signals.

**Table 2:** Structure of used derivate.

Chemical structures and properties				
Structure	Name of derivate	Molecular weight [g mol^−1^]	log*P*	Label
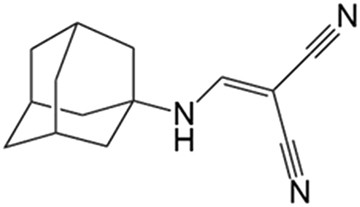	[(Tricyclo [3.3.1.1^3^, ^7^] dec-1-ylamino) methylidene] propanedinitrile	227.305	2.41 ± 0.75	E-A1
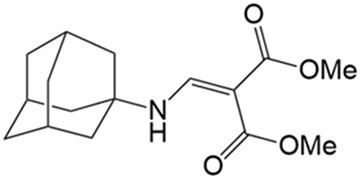	Dimethyl [(tricyclo [3.3.1.1^3^, ^7^] dec-1-ylamino) methylidene] propanedioate	293.316	2.47 ± 0.38	E-A2

Mass spectrometry was carried out using an Agilent 1260 LCMS system with APCI ionization in positive mode. Infrared (IR) spectra were recorded on a Perkin Elmer Spectrum Two spectrometer.

Gas chromatography (GC) analyses were performed using a Varian CX gas chromatograph equipped with a flame ionization detector (FID) to assess the purity of the compounds.

Detailed physicochemical characterization data, including experimental conditions and analytical results, are provided in the Supplementary Information (Sections S1–S3).

The compounds E-A1 and E-A2 were synthesized as described in the Supplementary Information (Section S4). Detailed experimental conditions and full analytical data are provided in the Supplementary Information.

#### 2.1.2 Other chemicals

DMEM:F12, FBS, RPMI-1640 with l-glutamine were purchased from Biosera (Cholet, France). GenElute™ Mammalian Genomic RNA Miniprep Kit, Gentamicin, HEPES, Hoechst 33342, JC-1, MTT ([3-(4, 5-dimethyldiazol-2-yl)-2, 5-diphenyltetrazolium bromide]), Penicillin-Streptomycin, primers, propidium iodide, tariquidar, gentamicine and vincristine were purchased from Merck, Slovakia (Bratislava, Slovakia). NaOH, NaCl, KCl, CaCl_2_ were purchased from Mikrochem (Pezinok, Slovakia). Tween 20 was purchased from MP Biomedicals (Santa Ana, California, USA).

Adamantane derivates E-A1 and E-A2 were purchased from Department of Organic Chemistry FCFT STU (Bratislava, Slovakia).

Annexin V FLUOS was purchased from Roche Diagnostics GmbH (Mannheim, Germany).

Calcein-AM, dNTP mix (10 mmol/l), Random Hexamer Primer (0.2 μg/μl), Reaction Buffer (5X), ReverAid H minus Reverse Transcriptase, RiboLock RNase Inhibitor, SYBr Green I Nucleic Gel Stain, Trypan blue, Ultrapure water were purchased from Invitrogen via Thermo Fisher Scientific (Waltham, MA, USA).

#### 2.1.3 Components of cell culture medium (CCM) and cultivation conditions

Human cell lines SKM-1 and MOLM-13 and their resistant variants SKM-1/VCR and MOLM-1/VCR were cultured in RPMI-1640 medium containing 16 % FBS and supplemented with penicillin-streptomycin solution (10,000 units of penicillin per 10 mg of streptomycin/1 ml). The adherent cell line H9C2 was cultured in DMEM medium supplemented with 10 % FBS. Variants of L1210 cells were cultured in 5 mL of RPMI-1640 medium supplemented with 8 % fetal bovine serum (FBS), penicillin-streptomycin solution (10,000 units of penicillin per 10 mg of streptomycin/1 ml) and 0.5 ng/mL gentamicin for 24 and 48 h at 37 °C in a humidified atmosphere containing 5 % CO_2_. Resistant R cells were periodically maintained by passaging every second passage in the presence of 250 nM vincristine. Prior to experiments, R cells were cultured for one passage in drug-free medium and subsequently used for further analysis. The expression of P-gp was periodically monitored at the mRNA level by RT-PCR.

The culture was carried out in a CO_2_ incubator at 37 °C and 5 % CO_2_ atmosphere. Suspension cells (5 × 10^5^ cells) were passaged every 48 h and adherent cells were passaged at 90 % confluence. Medium supplemented with 0.5 % DMSO was used as a control.

#### 2.1.4 PCR primers used

The primer sequences used in this study are shown in Table 3.

**Table 3:** PCR primers used.

Primer sequences for PCR			
Gene	Forward primer sequence (3′ → 5′)	Reverse primer sequence (5′ → 3′)	Base pair size
*ABCB1*	GCA​ATG​GAG​GAG​CAA​AGA​AG	CCA​AAG​TTC​CCA​CCA​CCA​TA	150
*ACTB*	GGC​ATG​GGT​CAG​AAG​GAT​T	AGG​TGT​GGT​GCC​AGA​TTT​C	133
*BAX*	CCC​CCG​AGA​GGT​CTT​TTT​CC	TAG​AAA​AGG​GCG​ACA​ACC​CG	84
*BCL-2*	GAA​CTG​GGG​GGA​GGA​TTG​TGG	GCC​GGT​TCA​GGT​ACT​CAG​TC	125
*TP53*	CCT​CCT​CAG​CAT​CTT​ATC​CG	TCA​TAG​GGC​ACC​ACC​ACA​CT	95

#### 2.1.5 Biological models

In the experimental part of this work, suspension cell lines of acute myeloid leukemia were used:

– SKM-1: Cell line isolated from the peripheral blood of a 76-year-old Japanese patient diagnosed with myelodysplastic syndrome, in whom the disease subsequently progressed to acute myeloid leukemia.
– MOLM-13: Cell culture from the blood of a 20-year-old man in whom AML developed from a previous myelodysplastic syndrome.

Both lines also had resistant variants: SKM-1/VCR and MOLM-13/VCR. These variants were obtained through long-term selection of parental cells in the presence of vincristine, which led to their adaptation to the cytotoxic effect of this drug. These variants were created by Institute of Molecular Physiology and Genetics, Center for Life Sciences, SAS [13].

Another line used was the H9C2 line – which is a subclone of the original clonal cell line derived from embryonic heart tissue (ventricular chamber) of the BD1X rat (*Rattus norvegicus*).

The AML suspension lines were provided by the Institute of Molecular Physiology and Genetics, Center for Life Sciences, SAS (originally obtained from the Leibniz-Institut, Deutsche Sammlung von Mikroorganismen und Zellkulturen GmbH, Braunschweig, Germany). The H9C2 adherent line was provided by the Institute for Heart Research, Center for Experimental Medicine, SAS.

Another cell model used in this study was the murine lymphoblastic leukemia cell line L1210 and its variants. The following variants of L1210 cells were used: (i) S, drug-sensitive parental cells obtained from the Leibniz-Institut DSMZ – Deutsche Sammlung von Mikroorganismen und Zellkulturen GmbH (Braunschweig, Germany) (ACC-123); (ii) R, P-gp-positive drug-resistant cells overexpressing murine P-glycoprotein as a result of long-term selection with vincristine; and (iii) T, P-gp-positive drug-resistant cells overexpressing human P-glycoprotein due to stable transfection with the P-gp gene from the Addgene plasmid 10957 (pHaMDRwt), a retroviral construct encoding full-length P-gp cDNA [14].

Cell viability was determined by trypan blue exclusion (4:1 dilution) using the Countess II FL (Thermo Fisher Scientific, USA) under aseptic conditions.

### 2.2 Methods

#### 2.2.1 MTT assay

The cytotoxic effect of amantadine derivatives E-A1 and E-A2 was determined using a colorimetric MTT assay. The 100 mM stock solution was diluted with DMSO to the desired final working concentrations (20 mM; 15 mM; 5 mM; 2 mM; 0.2 mM), which were subsequently used in further experiments. Cells (5 × 10^4^/200 µl) were incubated in 96-well microtiter plates in the absence or presence of derivatives (final concentration range of 1–100 µM) for 24 and 48 h. After the incubation time, the cells were centrifuged at 20 °C and 630 rcf for 10 min, and the supernatant was aspirated. MTT reagent (final concentration of 250 μg/ml) was added and the cells were further incubated for 3–4 h. The cells were incubated at 37 °C in the dark with gentle shaking. After the incubation time, the cells were centrifuged again (10 min, 20 °C, 900 rcf), the culture medium was aspirated, and 150 µl of 99.9 % DMSO was added to the cell pellet. In the last step, the absorbance at a wavelength of 540 nm was measured using a plate spectrophotometer (BioTech Instruments, Winooski, Vermont, USA).

#### 2.2.2 Detection of crystals by fluorescence microscopy

A suspension of cells of the leukemia cell line SKM-1 (6 × 10^6^ cells) was incubated in a Petri dish in the presence of the amantadine derivative E-A1 (*c* = 100 µM) for 24 h. After incubation, the suspension was quantitatively transferred to labeled Eppendorf microtubes, centrifuged (5 min, 500 rcf, 20 °C), the supernatant was removed, and 100 µl of serum-free RPMI-1640 medium without phenol red (BBM) was added to the pellet. This step was repeated twice. Finally, the cells were resuspended in 100 µl of BBM supplemented with Hoechst 33342 and visualized using a fluorescence microscope (Zeiss, Jena, Germany).

#### 2.2.3 Detection of cell death by flow cytometry

Human leukemia cell lines SKM-1 (/VCR) and MOLM-13 (/VCR) and murine leukemia cell lines L1210 (2.5 × 10^5^/500 µl) were cultured in a 24-well microtiter plates with the addition of the amantadine derivative E-A2 at four different concentrations (10, 25, 50 and 75 µM) for 24 and 48 h. After the incubation time, the cells were centrifuged for 5 min at 500 rcf and 20 °C. Subsequently, the cell pellet was resuspended and washed with serum-free RPMI-1640, and the cells were centrifuged under the same conditions. After removing the supernatant, the cell pellet was resuspended in 100 μl of binding buffer supplemented with annexin V-FITC (AV) at a final concentration of 0.5 μg/ml, while in the first control set only binding buffer without AV was used. The samples were incubated for 15 min in the dark at room temperature. Just before measurement, propidium iodide (PI) (0.1 μM) was added to the samples. In the final step, the percentage of cells in apoptosis and necrosis was analyzed using a BD Accuri C6 flow cytometer (BD Biosciences Pharmingen, San Diego, California, USA).

#### 2.2.4 Detection of P-glycoprotein transport activity by flow cytometry

AML cells (2.5 × 105/500 µl) were incubated for 24 and 48 h in a 24-well plate with the addition of the amantadine derivative E-A2 at four different concentrations (10, 15, 25 and 37.5 µM). After incubation, the cells were centrifuged (5 min, 500 rcf, 20 °C). Subsequently, the cell pellet was resuspended and washed with serum-free RPMI-1640, and the cells were centrifuged under the same conditions. As one of the controls for each cell line, a sample with the addition of tariquidar (TQR, *c* = 500 nM), a specific inhibitor of P-gp, was used. The cells were then suspended in 100 μl of serum-free RPMI-1640 and incubated in the dark on a shaker at 37 °C with constant stirring for approximately 1 h. After the selected time, calcein-AM was added to all samples (except for the first set of controls) to a final concentration of 10 nM and the mixture was incubated again in the dark for approximately 10 min. The mixture was centrifuged again and washed twice in 100 μl of serum-free RPMI-1640. Finally, PI (0.1 μM) was added to the samples just before measurement and the mixture was analyzed using a BD Accuri C6 flow cytometer.

#### 2.2.5 qPCR

AML cells were incubated for 24 h in the presence of the amantadine derivative E-A2 (10, 25, and 37.5 μM). After centrifugation (5 min, 500 rcf, 20 °C), the pellet was resuspended in 300 µl of sterile deionized water and transferred to an Eppendorf tube. Then, 300 µl of TRI reagent was added. After vortexing, the samples were incubated for 5 min at room temperature. Subsequently, 100 µl of chloroform was added, the samples were mixed, and incubated for another 10 min. Centrifugation followed (15 min, 3,000 rcf, 4 °C). The upper aqueous phase was transferred to a new tube, and RNA was precipitated using 250 µl of isopropanol (−20 °C). After 10 min of incubation and centrifugation, the RNA pellet was washed with 200 µl of ethanol, centrifuged again in the same conditions, the supernatant was removed, and the pellet was allowed to air-dry. RNA was resuspended in 30 µl of ultrapure water, and its concentration was measured spectrophotometrically.

From the obtained RNA (1,000 ng), cDNA synthesis was prepared by adding hexamer primers (0.2 μg/μl) and ultrapure water up to a volume of 12 µl. Then, a reaction mixture containing reverse transcriptase, buffer, and dNTPs was added. Reverse transcription was carried out in a thermal cycler (Eppendorf, Hamburg, Germany) at 42 °C for 60 min, then at 70 °C for 10 min, followed by cooling to 4 °C. The cDNA samples were stored at −20 °C.

The reaction mix for Real-Time PCR was prepared on a chilled rack. For each well of the 96-well plate, 1 µl of cDNA and a MasterMix with the appropriate primers were added. PCR was carried out under the following conditions: denaturation (95 °C, 10 s), annealing and elongation (60 or 57 °C, 30 s), and termination (65 °C, 5 s and 95 °C, 5 s). After the PCR run, samples were stored at −20 °C. The primers used were specific for the genes *ABCB1, ACTB, BAX, BCL-2*, and *TP53*, and the size of the resulting amplicons ranged from 84 to 150 base pairs. The primer sequences and expected amplicon sizes are listed in Table 3 PCR primer used.

**Research ethics:** Not applicable.

**Informed consent:** Not applicable.

## 3 Results

### 3.1 Cytotoxicity assessment of amantadine derivatives

**Figure 1:**
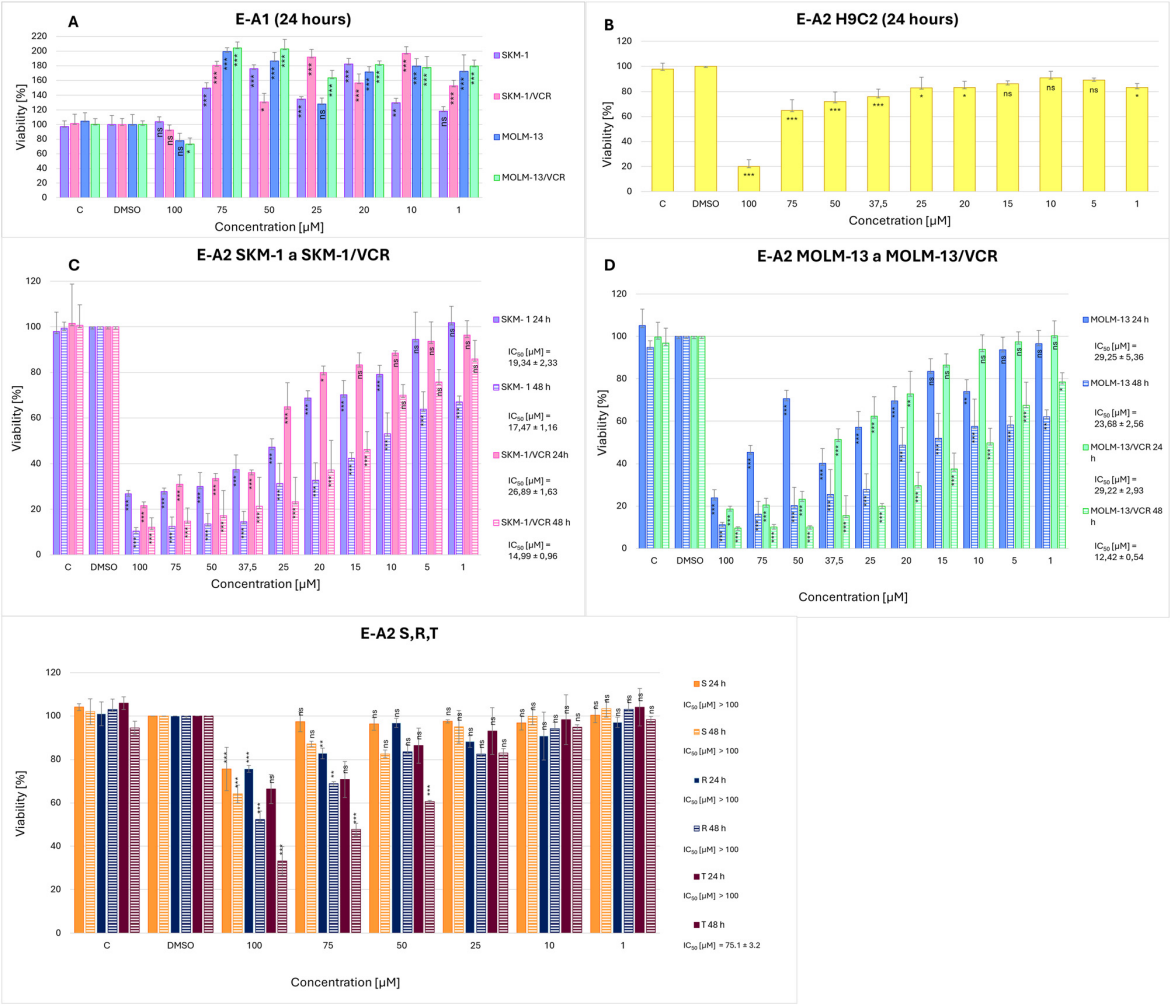
Cytotoxicity by MTT. Cells were cultured for 24 (A–D) and 48 h (A,C,D) in the presence or absence of E-A1 (A) and E-A2 (B–E) derivatives at the indicated concentrations. The level of statistical significance was determined using one-way analysis of variance (ANOVA). Probability values were interpreted as follows: *p* > 0.05 (non-significant – ns), *p* < 0.05 (* – marginally significant), *p* < 0.01 (** – moderately significant) and *p* < 0.001 (*** – highly significant).

**Figure 2:**
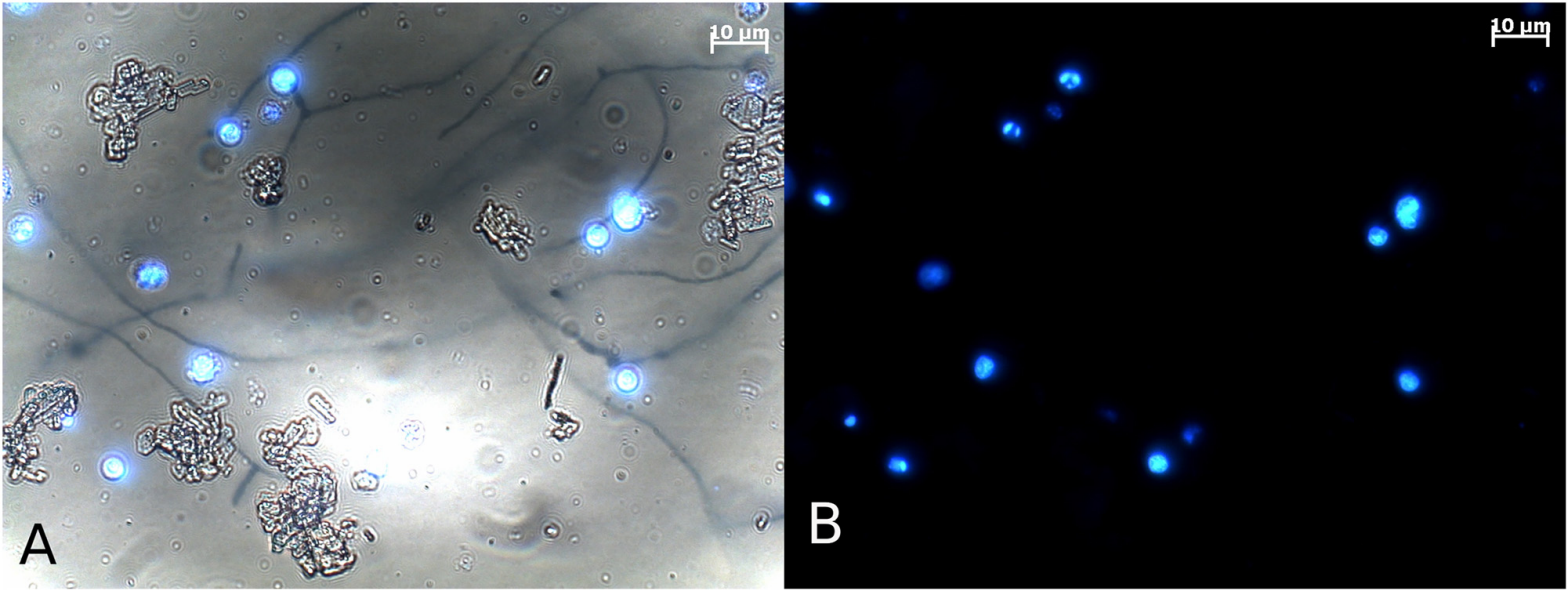
Visualization of the formed crystals of the E-A1 derivative using fluorescence microscopy SKM-1 cells are labeled with the Hoechst33342 probe (blue). Total magnification 400×. A: Combined imaging in light (bright field) and fluorescence mode. Crystals and fluorescently labeled nuclei (blue signals) are visible in the sample. B: Imaging in fluorescence mode. Only specifically labeled cell nuclei are detected against a dark background.

In the initial experiment, we tested the cytotoxicity of the E-A1 derivative on leukemia cell lines SKM-1, MOLM-13, and their resistant variants. After a 24-h incubation with various concentrations (1–100 μM), no cytotoxic effect was observed; in fact, cell viability often exceeded that of the control. The most significant increase (203 %) was seen at 75 μM in the resistant MOLM-13 line (Figure 1A). Based on these results, E-A1 was further examined by fluorescence microscopy, while subsequent cytotoxicity studies focused on a different derivative, E-A2 (Figure 2).

E-A2 was tested on the same leukemia cell lines and their vincristine-resistant variants (Figure 1C-D). Ten concentrations (1–100 μM) were used, with 24- and 48-h incubations. The most pronounced cytotoxic effect was observed in the SKM-1 line and its resistant variant, where viability dropped below 40 % at higher concentrations, and down to 10 %–22 % after 48 h. MOLM-13 showed lower sensitivity overall, though its resistant variant exhibited a more noticeable reduction in metabolic activity at higher E-A2 concentrations.

Since the derivative was initially tested only on acute myeloid leukemia cells, we also examined its effect on healthy cells. We selected the rat cardiomyocyte cell line H9C2. In this case, no significant cytotoxic effect was observed; only the highest concentration had a noticeable impact (Figure 1B).

To further support the observed effects, the activity of compound E-A2 was evaluated in an additional leukemia model using L1210 cells and their variants (S, R, and T) (Figure 1E). The compound was tested at concentrations ranging from 1 to 100 µM for 24 and 48 h.

In the drug-sensitive S cells, E-A2 exhibited only a moderate effect after 24 h, with a more pronounced decrease in cell viability observed at 48 h, particularly at higher concentrations.

In contrast, resistant R cells showed a stronger reduction in viability, especially after 48 h of incubation, where a clear concentration-dependent effect was observed, with the highest concentration reducing viability to approximately 50 %.

The most pronounced effect was observed in T cells expressing human P-gp, where E-A2 induced a substantial decrease in cell viability in a time- and concentration-dependent manner. After 48 h, viability was reduced to approximately 33 % at 100 µM and below 50 % already at 75 µM.

These findings are in agreement with the results obtained in human AML cell lines, where comparable or even lower IC_50_ values were observed in resistant variants compared to their parental counterparts (e.g., MOLM-13/VCR: 12 µM vs. MOLM-13: 24 µM after 48 h).

Overall, these results indicate that E-A2 retains its cytotoxic activity in P-gp-expressing cells and is not significantly affected by P-gp-mediated drug resistance.

### 3.2 Crystal detection using fluorescence microscopy

Based on the results obtained from the MTT assay, we decided to further examine the amantadine derivative E-A1 using fluorescence microscopy. The SKM-1 cell line was incubated with E-A1 (*c* = 100 μM) for 24 h. For visualization, we used fluorescent staining with the Hoechst 33342 probe, which enabled us to detect the formation of E-A1 crystals. As shown in the representative images, the presence of crystals in the E-A1 sample was successfully captured and visualized. The nuclei of SKM-1 cells were stained with the Hoechst 33342 fluorescent probe and appear in blue.

Crystallization of the compound likely led to a reduction in the effective concentration of the active substance in the solution, which may explain the lack of effect observed in the treated cell lines. Due to crystal sedimentation, the availability of the compound to the cells was probably significantly limited. For this reason, we decided not to use this derivative in further experiments (Figure 2).

### 3.3 Detection of cell death using flow cytometry

**Figure 3:**
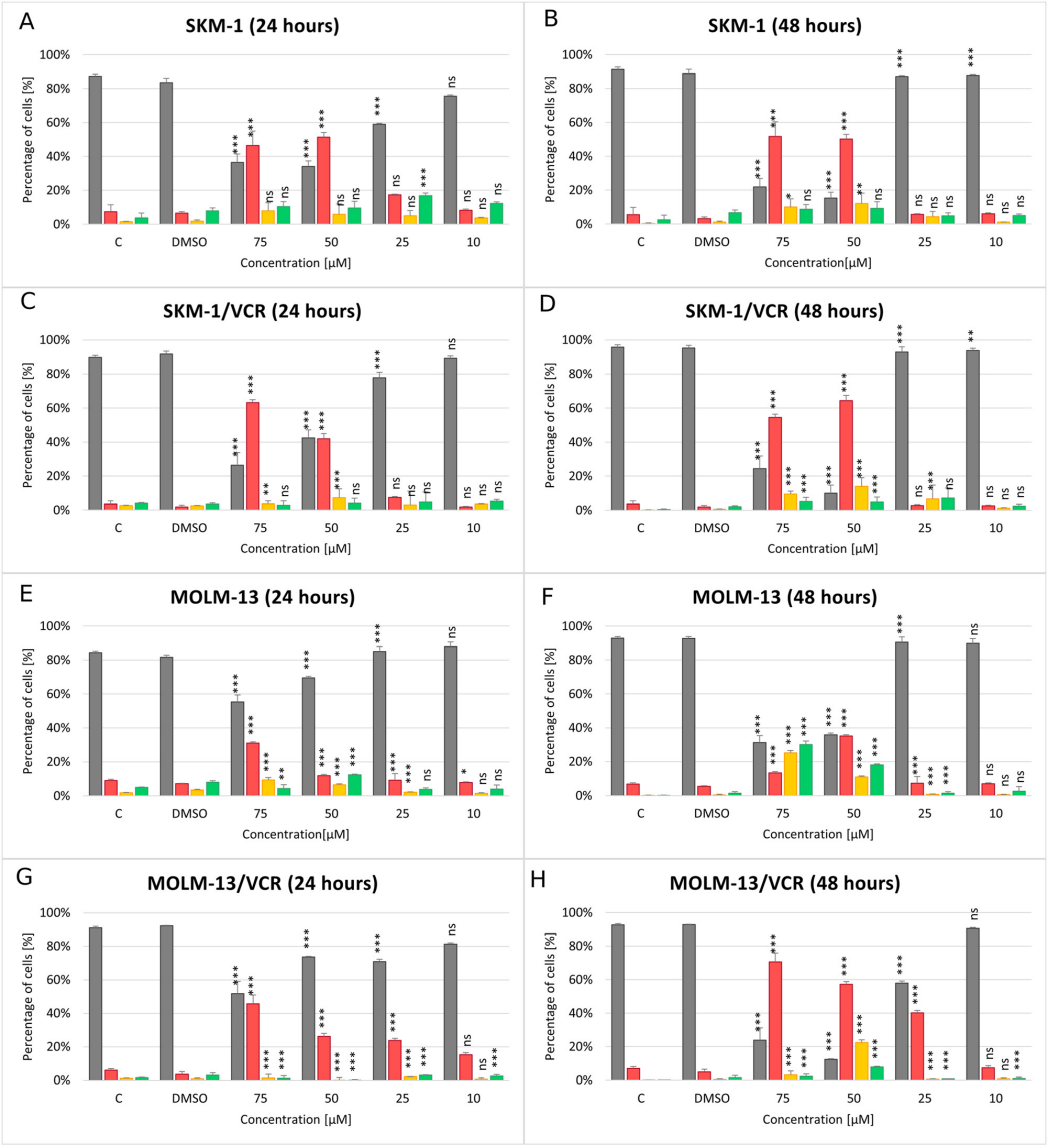

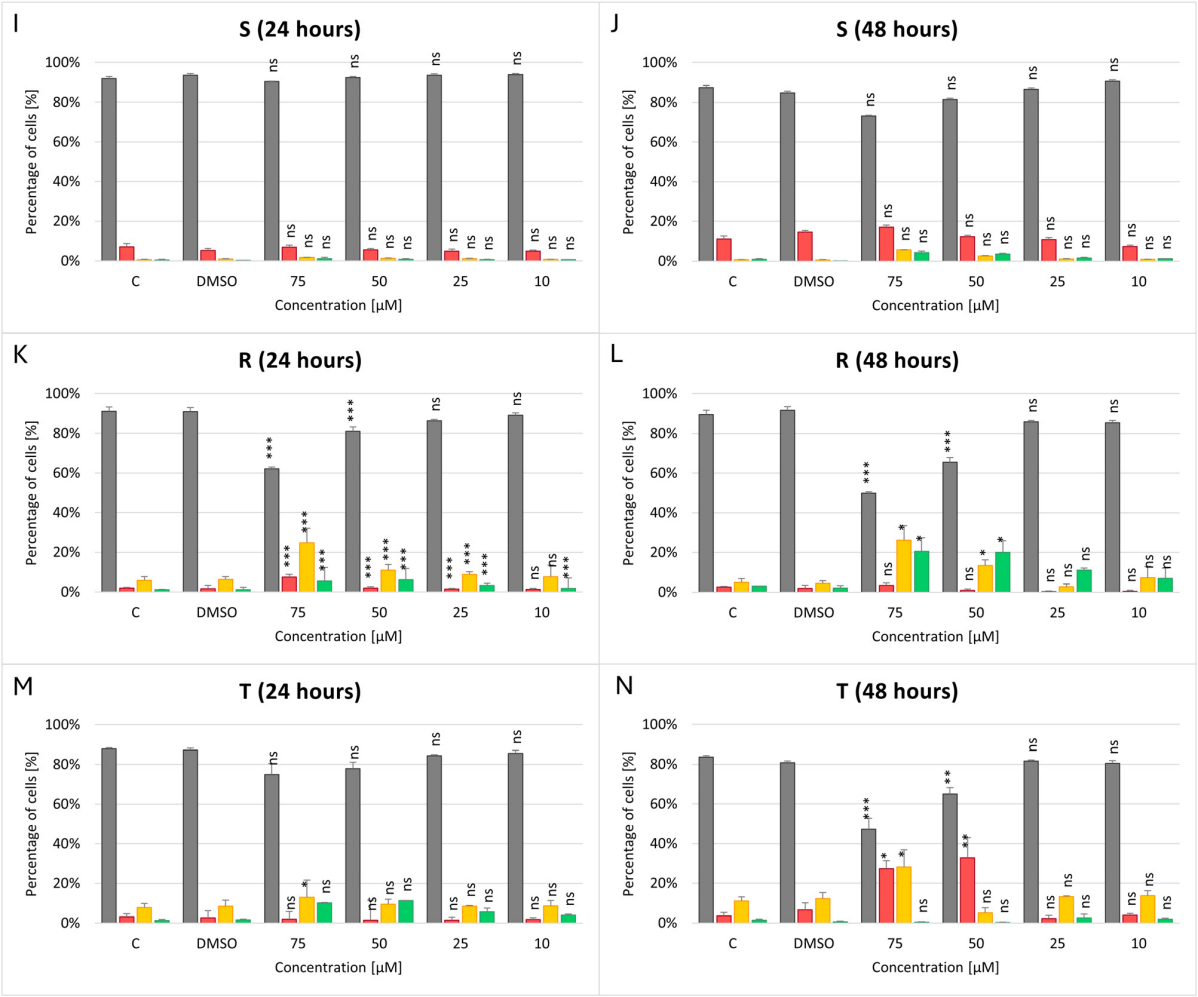
Determination of cell viability by the AV/PI assay. Cells were cultured for 24 (A, C, E, G, I, K, M) and 48 h (B, D, F, G, J, L, N) in the presence or absence of E-A2 derivative at the indicated concentrations. Legends gray: viable cells, red: necrotic cells, yellow: cells in late apoptosis, green: apoptotic cells. The level of statistical significance was determined using one-way analysis of variance (ANOVA). Probability values were interpreted as follows: *p* > 0.05 (non-significant – ns), *p* < 0.05 (* – marginally significant), *p* < 0.01 (** – moderately significant) and *p* < 0.001 (*** – highly significant).

We investigated the type of cell death using the procedure described in the relevant subsection (Section 3.2.4). Four variants of cell lines – SKM-1, MOLM-13, and their resistant counterparts (SKM-1/VCR, MOLM-13/VCR) – were incubated for 24 and 48 h in the presence of the amantadine derivative E-A2 at four different micromolar concentrations: 10, 25, 50, and 75 μM (Figure 3A-H). To determine the type of cell death, we used fluorescent probes. Fluorescein isothiocyanate-labeled AV is used to identify the externalization of phosphatidylserine, a defining feature of apoptosis. Necrotic cells are detected using propidium iodide (PI), a DNA-intercalating dye that cannot pass through intact plasma membranes and therefore stains cells with compromised membrane integrity selectively. The results were obtained using a BD Accuri C6 flow cytometer and are presented graphically.

The greatest decrease in viability was observed in the resistant variant of the SKM-1 cell line, where cell viability at higher concentrations (75, 50 μM) ranged from 26 % to 43 % after 24 h and from 9 % to 25 % after 48 h of E-A2 treatment. In the non-resistant SKM-1 variant, cells were more viable following E-A2 exposure, with viability of approximately 35 % after 24 h and 18 % after 48 h (again at the higher concentrations). At lower concentrations (25, 10 μM), the difference in viability compared to the controls was less noticeable. The most prevalent type of cell death in the SKM-1 line and its resistant variant was necrosis.

In the MOLM-13 cell line, the differences in cell viability were less distinct compared to those in SKM-1. After 24 h of E-A2 treatment, viability dropped by approximately 30 % compared to the control (at 75 and 50 μM), and by about 60 % after 48 h. The resistant variant of the MOLM-13 line was slightly more sensitive to E-A2, particularly after 48 h, when viability ranged from 12 %–24 % (*c* = 75, 50 μM). After 24 h, only minor differences were observed between the resistant and non-resistant variants of the MOLM-13 line. As with the SKM-1 line, lower concentrations (25, 10 μM) showed little difference from the control samples. In both MOLM-13 and MOLM-13/VCR lines, the predominant form of cell death was again necrosis.

To further characterize the mode of cell death induced by E-A2, AV/PI staining followed by flow cytometry analysis was performed in L1210 cells and their variants (S, R, and T) after 24 and 48 h of treatment (Figure 3I-N).

After 24 h of incubation, only minor changes in the distribution of cell populations were observed across all variants. The majority of cells remained viable, with only a slight increase in early and late apoptotic populations at higher concentrations, indicating a limited early effect of E-A2 on apoptosis induction.

In contrast, after 48 h of treatment, a pronounced shift in cell populations was detected, particularly at higher concentrations (75 and 50 µM). In S cells, the proportion of viable cells decreased (to ∼73 % at 75 µM), accompanied by an increase in both early and late apoptotic populations.

A more substantial effect was observed in resistant R cells, where treatment with 75 µM reduced the viable population to approximately 50 %, with a marked increase in late apoptosis (∼26 %) and early apoptosis (∼21 %). Similar but slightly less pronounced changes were observed at 50 µM.

In T cells expressing human P-gp, E-A2 also induced apoptosis, although the response was more variable. Nevertheless, a decrease in viable cells and an increase in apoptotic populations were observed, supporting the ability of E-A2 to induce cell death even in cells expressing human P-gp.

Overall, these results indicate that the cytotoxic effect of E-A2 is at least partially mediated by the induction of apoptosis in a time- and concentration-dependent manner, with a more pronounced effect observed after 48 h of treatment.

Representative flow cytometry dot plots are provided in the Supplementary Information (Figures S18 and 19).

### 3.4 Detection of P-gp transport activity using flow cytometry

P-gp transport activity was quantified using a BD Accuri C6 flow cytometer, using calcein acetoxymethyl ester (calcein/AM) as a specific P-gp substrate. In this experiment, a specific P-gp inhibitor, tariquidar, was used as a positive control.

**Figure 4:**
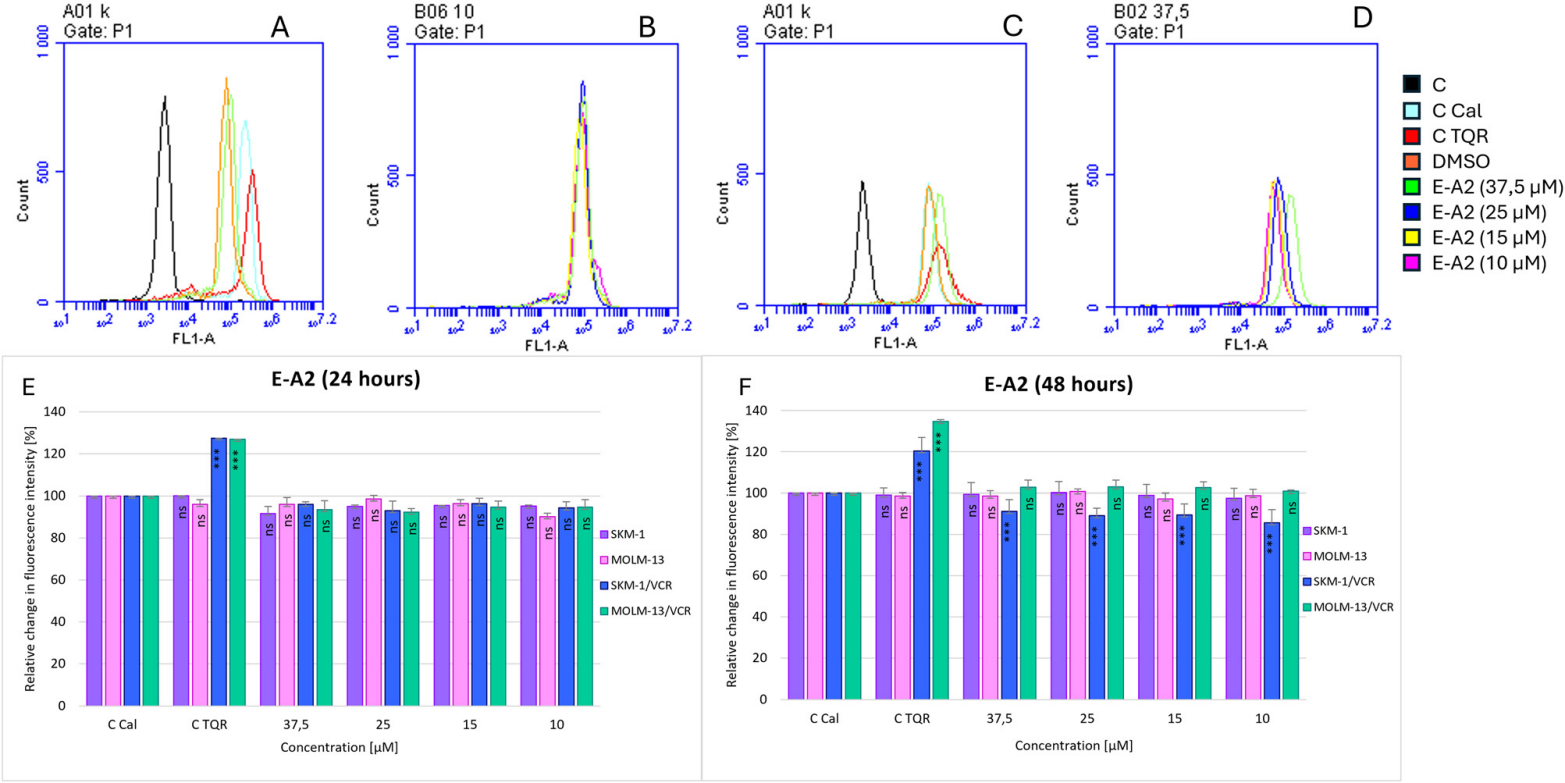
Determination of calcein retention. Cells were cultured for 24 (E) and 48 h (A–D, F) in the presence or absence of E-A2 derivative at the indicated concentrations. Sample histograms of calcein retention on the SKM-1 cell line (A–B) and MOLM-13 cell line (C–D) after 48 h incubation. Colored curves represent: purple – control without TQR addition; red – control with TQR addition; orange – control with DMSO without TQR addition; green – *c* = 37.5 μM; blue – *c* = 25 μM; yellow – *c* = 15 μM; pink – *c* = 10 μM. The level of statistical significance was determined using one-way analysis of variance (ANOVA). Probability values were interpreted as follows: *p* > 0.05 (non-significant – ns), *p* < 0.05 (* – marginally significant), *p* < 0.01 (** – moderately significant) and *p* < 0.001 (*** – highly significant).

The values shown in the graphs represent the median fluorescence intensity ratios normalized to the calcein/AM-labeled control Figure 4. As can be seen from the graphs, the tested derivative was not capable of inhibiting P-gp, as the fluorescence intensity changed only slightly across all concentrations. The graphs also show that there were no significant differences in the change of fluorescence intensity between individual lines (non-resistant vs. resistant lines) after 24 and 48 h. The highest values of fluorescence intensity change are observed among all cell lines in the MOLM-13/VCR line. The sample histograms represent the SKM-1 and SKM/VCR cell lines after 48 h of treatment with the E-A2 derivative. It is clear from the visualization that the curves closely overlap, which again indicates that the given derivative did not induce the activity of the monitored pump in non-resistant variants of AML cells.

### 3.5 qPCR

The last analysis performed in this work was quantitative polymerase chain reaction (qPCR) of selected genes in acute myeloid leukemia cells (SKM-1, SKM-1/VCR, MOLM-13, MOLM-13/VCR) after 24 h of treatment with the E-A2 derivative (*c* = 10, 25 and 37.5 μM).

**Figure 5:**
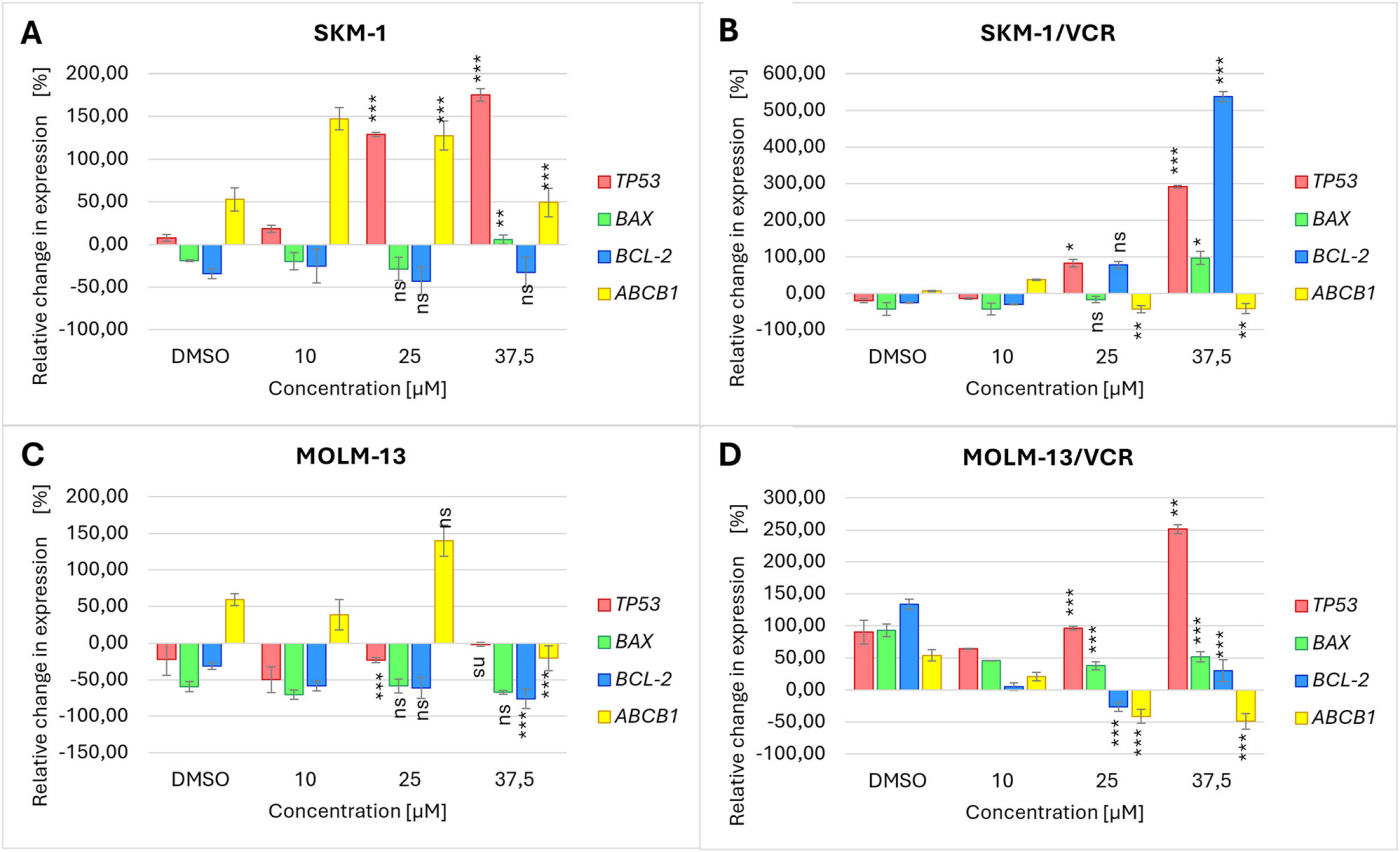
Expression of selected genes: TP53, BCL-2,BAX and ABCB1. The results are representative of three independent measurements of triplicate experiments, and ACTB was used as a housekeeper. Cells were cultured for 24 h in the presence or absence of E-A2 derivative at the indicated concentrations. The level of statistical significance was determined using one-way analysis of variance (ANOVA). Probability values were interpreted as follows: *p* > 0.05 (non-significant – ns), *p* < 0.05 (* – marginally significant), *p* < 0.01 (** – moderately significant) and *p* < 0.001 (*** – highly significant).

We chose the following apoptotic genes as target markers due to their key role in the regulation of programmed cell death: *TP53* as a cell cycle regulator and apoptosis trigger; *BAX* as a pro-apoptotic and *BCL-2* as an anti-apoptotic gene, which together determine the “decision” of the cell between survival and death. *ABCB1* (P-gp), a gene encoding a transporter associated with drug efflux, was included due to its role in the development of resistance. *ACTB* (β-actin) was used as a reference (control) gene, which is stably expressed in cells (Figure 5).

In the SKM-1 cell line, we observed the largest changes in the expression of the genes encoding *TP53* and *ABCB1*. The relative change in expression showed a concentration-dependent pattern for *TP53*. At the highest concentration (37.5 μM) we observed an increase of over 175 %. The highest change in relative expression was observed in the gene encoding *ABCB1* at a concentration of 10 μM (+147 %). In other genes, the value of relative expression decreased or increased only slightly (*BAX*, *c* = 37.5 μM, increase by 5 %).

In the resistant variant SKM-1/VCR, we observed the largest difference in the gene encoding *BCL-2* (*c* = 37.5 μM), in which the value of relative expression increased by almost 540 %. At the highest concentration, there was an increase in expression of the gene encoding *TP53* (+290 %) and *BAX* (+97 %). At lower concentrations, the changes in gene expression were less pronounced.

In the MOLM-13 cell line, a negative change in relative expression predominated; for most genes, the relative expression decreased compared to the control. An increase is observed only in the gene encoding *ABCB1*, for which the highest change occurred at a concentration of 25 μM (+140 %).

The MOLM-13/VCR cell line showed the most pronounced changes in expression of the gene encoding *TP53*, again at the highest concentration (+250 %). In the other samples, the changes were less significant.

Finally, we decided to evaluate the *BAX/BCL-2* ratio, which shows the balance between anti-apoptotic and pro-apoptotic mechanisms. If the ratio is >1 → predominance of pro-apoptotic processes, ratio ≈ 1 → equilibrium, ratio < 1 → predominance of anti-apoptotic processes. Table 4 summarizes the obtained results.

**Table 4:** *BAX/BCL*-*2* ratio.

Relative expression/activity in cell lines					
Cell line	*K*	DMSO	10 µM	25 µM	37.5 µM
SKM-1	1.00	1.23	1.08	1.25	1.57
SKM-1/VCR	1.00	0.77	0.82	0.47	0.31
MOLM-13	1.00	0.59	0.71	1.07	1.37
MOLM-13/VCR	1.00	0.83	1.39	1.87	1.16

The results show that the balance between the relative expression of the gene encoding *BAX* to the gene encoding *BCL-2* is clearly shifted towards anti-apoptotic processes only in the SKM-1/VCR line. Its non-resistant variant shows the opposite result – the ratio across all concentrations indicates a shift towards pro-apoptotic processes. For the MOLM-13 line and its resistant variant, the results are mixed – however, higher concentrations indicate a stronger occurrence of involvement of pro-apoptotic pathways.

## 4 Discussion

At the beginning of the experimental work, the MTT assay was employed to evaluate the cytotoxic effects of amantadine derivatives (E-A1 and E-A2) after 24 and 48 h of incubation on acute myeloid leukemia cell lines (SKM-1, SKM-1/VCR, MOLM-13, MOLM-13/VCR). Both derivatives share the same core structure – adamantylamine – but differ due to the attachment of distinct electrophilic reagents.

The results indicated that the E-A1 derivative did not exert a significant cytotoxic effect on the tested cell lines (Figure 1). In most cases, cell viability remained unchanged or even increased compared to the control group. Notably, in the resistant MOLM-13/VCR variant, treatment with 75 μM E-A1 resulted in an increase in cell viability to 203 %. This suggests that E-A1 may not be cytotoxic but could stimulate cellular processes related to enhanced metabolic activity or proliferation, such as mitochondrial stimulation or activation of growth factor pathways [15], 16].

However, interpretation of these results must consider the physicochemical properties of E-A1, which may have influenced its bioavailability in the experimental setup. During solution preparation, we observed that E-A1 exhibited poor solubility in commonly used solvents, including DMSO, and tended to crystallize despite thorough mixing and short-term incubation at elevated temperatures. This crystallization likely reduced the effective concentration of the derivative in the culture medium.

To verify this, fluorescence microscopy was performed, confirming the presence of crystals (Figure 2). We therefore conclude that limited cellular penetration of E-A1 contributed to its reduced cytotoxic activity. Based on these findings, subsequent experiments were conducted exclusively with the E-A2 derivative.

The cytotoxic effect of the E-A2 derivative was most pronounced in the SKM-1 cell line and its resistant variant. At the highest tested concentrations (100, 75, 50, and 37.5 μM), cell viability significantly decreased, reaching values between 10 % and 22 % after 48 h of incubation. In contrast, the MOLM-13 cell line and its resistant variant exhibited a less pronounced cytotoxic response, although a significant reduction in metabolic activity was still observed at higher concentrations (Figure 1).

To further support these findings, the effect of E-A2 was evaluated in an additional leukemia model using L1210 cells and their variants (S, R, and T). A time- and concentration-dependent decrease in viability was observed, with the strongest effect detected in T cells expressing human P-gp. After 48 h, cell viability decreased to approximately 33 % at 100 µM and below 50 % at 75 µM. These results are consistent with the observations in human AML cell lines and further indicate that E-A2 retains its cytotoxic activity in P-gp-expressing cells.

Despite both derivatives originating from the same starting material, their cytotoxic effects differed markedly. This can be explained by differences in their chemical structures: the E-A1 derivative contains two nitrile groups within a relatively nonpolar and rigid conjugated system. This structural feature likely contributes to its poor solubility (as confirmed experimentally), reduced cellular penetration, and limited interaction with biological targets [17]. Conversely, the E-A2 derivative possesses three ester groups introduced during synthesis, which increase the molecule’s overall hydrophilicity and solubility in the culture medium, ensuring a higher effective concentration available to cells [18]. Enhanced solubility also increases the probability of cellular membrane penetration and subsequent intracellular activity. Chemically, the ester groups in E-A2 may undergo hydrolysis, potentially generating more active metabolites that further contribute to its cytotoxicity [19].

Before assessing cell death, we evaluated the cytotoxicity of the E-A2 derivative on healthy, non-malignant cells (Figure 1). As a model, we used the rat embryonic cardiomyocyte cell line H9C2, given that adverse cardiac effects of amantadine have been reported since the 1970s [20]. Interestingly, in contrast to the pronounced cytotoxicity observed in leukemic cell lines, E-A2 showed no significant toxicity to healthy cardiac cells, with a marked effect detected only at the highest concentration (100 μM). This difference in sensitivity may be influenced by several factors. Leukemic cells typically display increased metabolic activity, a higher proportion of proliferating cells, and dysregulated apoptotic pathways, making them more susceptible to cytotoxic agents. In contrast, healthy cells proliferate more slowly and possess more robust protective mechanisms, potentially rendering them less sensitive to E-A2. Furthermore, variations in the expression of transport proteins, molecular targets, or drug-metabolizing enzymes could also underlie the preferential activity of the derivative against malignant cells while sparing healthy ones [21], [22], [23]. Based on the MTT assay results, we conclude that the E-A2 derivative effectively suppresses metabolic activity and cell proliferation. Therefore, we proceeded to investigate the specific mode of cell death induced by this compound in leukemic cell lines and their resistant variants.

We analyzed cell death in leukemia cell lines after 24- and 48-h exposure to E-A2 (10–75 μM) (Figure 3). The most pronounced reduction in viability was observed in SKM-1/VCR cells, followed by SKM-1, MOLM-13/VCR, and MOLM-13, with minimal effects at lower concentrations (10 and 25 μM). These findings are consistent with the MTT results, which demonstrated comparable or even increased sensitivity of resistant variants relative to their parental counterparts.

Flow cytometry analysis revealed that cell death was time- and concentration-dependent, with a more pronounced effect after 48 h of treatment. While necrotic cell populations were prominent, particularly at higher concentrations, a clear increase in both early and late apoptotic populations was also observed. This indicates that E-A2 induces a mixed mode of cell death, involving both apoptotic and necrotic processes.

Importantly, similar trends were confirmed in the L1210 model, including variants expressing either murine or human P-gp. The maintained or even enhanced sensitivity of these cells suggests that E-A2 is not effectively effluxed by P-gp and may partially overcome P-gp-mediated drug resistance.

Taken together, these findings support the potential of E-A2 as a compound capable of inducing cell death in both sensitive and multidrug-resistant leukemia cells, likely through mechanisms that are not significantly limited by P-gp activity.

To further assess P-gp involvement, we measured calcein retention to determine whether P-gp activity in resistant cell lines influences intracellular E-A2 concentration and, consequently, the mode of cell death. Despite resistance and P-gp expression, necrosis remained high, prompting this analysis. Calcein/AM measurements showed that E-A2 did not inhibit P-gp: fluorescence intensity changed minimally across all concentrations (10, 15, 25, 37.5 μM) and cell lines (SKM-1, SKM-1/VCR, MOLM-13, MOLM-13/VCR) (Figure 4). No significant differences were observed between resistant and non-resistant lines, indicating no effect on P-gp function even with elevated expression.

In contrast, the positive control tariquidar shifted the fluorescence signal (FL1-A) in resistant lines, confirming both its inhibitory activity and assay validity. Histograms (Figure 4A–D) clearly show this shift for SKM-1 and MOLM-13. These results suggest that E-A2 is unlikely to be a P-gp substrate. If it were, changes in fluorescence would be expected in resistant lines – either decreased (competition for binding) or increased (temporary transporter blockade). The absence of such changes indicates that E-A2 likely does not interact with P-gp as a substrate or inhibitor [24], 25].

Regarding the predominant cell death type, calcein retention results suggest that if E-A2 is not a P-gp substrate, its intracellular concentration may reach toxic levels in both resistant and non-resistant lines, regardless of P-gp presence. The lack of apoptosis may indicate that E-A2 does not activate classical apoptotic pathways (e.g., the caspase cascade), possibly due to its chemical structure derived from amantadine [26], 27].

At the end of the experimental part, changes in gene expression were analyzed using quantitative real-time PCR (Figure 5). We examined *TP53, BAX, BCL-2*, and *ABCB1*, with *ACTB* as the reference gene. The most notable changes were in *TP53*, a central regulator of the cell cycle and apoptosis, which showed strong induction after E-A2 exposure, particularly at 37.5 μM (up to +290% in SKM-1/VCR and +250% in MOLM-13/VCR), indicating activation of stress responses and potential apoptotic pathways in resistant cells. Non-resistant SKM-1 showed a similar but milder concentration-dependent trend.

In *ABCB1,* non-resistant SKM-1 and MOLM-13 displayed increased expression mainly at lower E-A2 concentrations (10 and 25 μM), likely reflecting an adaptive xenobiotic response. In resistant SKM-1/VCR, BCL-2 expression rose sharply (+540 % at 37.5 μM), consistent with reinforced anti-apoptotic defense, yet *BAX* also increased, suggesting a regulatory balance between survival and death signals. Evaluating the *BAX/BCL-2* ratio revealed a shift towards anti-apoptosis (ratio < 1) in SKM-1/VCR, matching prior findings of low apoptosis in this line, whereas the non-resistant variant maintained a pro-apoptotic profile (ratio > 1) (Table 4). MOLM-13 and MOLM-13/VCR showed more variable results, but higher E-A2 concentrations generally favored pro-apoptotic responses.

## 5 Conclusions

The cytotoxic effects of amantadine derivatives E-A1 and E-A2 were assessed on AML cell lines SKM-1, MOLM-13, their resistant variants, and healthy rat cardiomyocytes (H9C2) using MTT assays. E-A1 showed negligible cytotoxicity, likely due to poor solubility, crystal formation, and limited permeability. In contrast, E-A2 was highly cytotoxic, especially in SKM-1 and SKM-1/VCR cells, correlating with improved solubility and possible metabolite formation, while sparing H9C2 cells. Flow cytometry revealed predominantly necrotic cell death, particularly in SKM-1/VCR at higher concentrations. The results obtained from MTT and flow cytometry analyses in the L1210 model further confirmed that E-A2 exerts a time- and concentration-dependent cytotoxic effect, including in variants expressing murine and human P-gp. Calcein-AM assays confirmed no P-gp inhibition or substrate activity, with P-gp expression limited to resistant lines. qRT-PCR showed *TP53* upregulation, especially in resistant lines, and increased *BCL-*2 and *BAX* in SKM-1/VCR, indicating complex apoptotic regulation. The *BAX/BCL-2* ratio suggested an anti-apoptotic profile in resistant versus pro-apoptotic in non-resistant cells. Low E-A2 concentrations induced *ABCB1* in non-resistant cells, implying an adaptive xenobiotic response. Overall, E-A2 emerges as a selective AML cytotoxic agent acting mainly via necrosis and stress-mediated pathways, independent of P-gp modulation. A limitation of this study is the absence of detailed mechanistic analyses at the molecular level, which will be the focus of future investigations.

## Supplementary Material

Supplementary Material
