# Supplementary material for: Cellular effects of adamantane derivatives in P-glycoprotein leukemia cells
Source: Open Life Sci. 2026 Jun 26;21(1):20251340. doi: 10.1515/biol-2025-1340 (PMC13296374; doi:10.1515/biol-2025-1340)
Supplement: Supplementary file 1 — Supplementary Material [file j_biol-2025-1340_suppl_001.docx]

**Supplementary Information**

**S.1 Chemical characterization of E-A1 and E-A2**

**S1.1 Instrumentation**

Melting points were determined using a Boetius apparatus (Nagema, Radebeul, Germany) equipped with a high-precision thermometer (TD 121, VWR) and are uncorrected.

Nuclear magnetic resonance (NMR) spectra were recorded on a Varian Unity Inova 300 MHz spectrometer (300 MHz for ¹H and 75 MHz for ¹³C) equipped with an H/F/X ATB broadband probe. Chemical shifts (δ) are reported in parts per million (ppm) and were referenced to tetramethylsilane (TMS) as an internal standard. Residual solvent signals were used as secondary references when appropriate (¹H: CHCl₃, 7.16 ppm; DMSO-d₆, 2.50 ppm; ¹³C: CDCl₃, 77.16 ppm; DMSO-d₆, 39.52 ppm).

Mass spectrometry (MS) measurements were performed using an Agilent 1260 LCMS system equipped with a single quadrupole mass detector and a multimode ionization source (ESI/APCI). Spectra were acquired in positive APCI mode. Samples were introduced as methanolic solutions (1 mg/mL) and delivered via a stainless-steel capillary at a flow rate of 0.4 mL/min.

Infrared (IR) spectra were recorded on a PerkinElmer Spectrum Two spectrometer. Wavenumbers are reported in cm⁻¹.

Gas chromatography (GC) analyses were carried out using a Varian CX gas chromatograph (S/N: 03918750-32) equipped with a flame ionization detector (FID). Separation was achieved on an SGE Analytical Science BP10 capillary column (30 m × 0.25 mm i.d., 0.25 μm film thickness) using nitrogen as the carrier gas at a constant pressure corresponding to an initial flow rate of 2.0 mL/min. Injector and detector temperatures were maintained at 320 °C. Samples were injected with a split ratio of 1:20.

The oven temperature programs were as follows:
For E-A1: initial temperature 50 °C (2 min), increased to 100 °C at 50 °C/min, then to 280 °C at 20 °C/min (4 min hold), and finally to 300 °C at 6 °C/min (total run time 19.33 min).
For E-A2: initial temperature 50 °C, increased to 100 °C at 50 °C/min (2 min hold), then to 280 °C at 50 °C/min (15 min hold), and finally to 300 °C at 6 °C/min (total run time 24.93 min).

**S1.2. Compound E-A1**

**Melting point:** The compound did not exhibit a clear melting point up to 280 °C and showed no visible decomposition. A gradual mass loss was observed above 200 °C, which may indicate partial sublimation.

**¹³C NMR (75 MHz, DMSO-d₆):** δ 156.29, 117.13, 54.85, 46.10, 41.12, 35.00, 28.59. Signals corresponding to carbons C14 and C15 appear very close in chemical shift, which may be attributed to rapid double bond isomerization within a push–pull system. **(**Figure S1; Figure S2)

**¹H NMR (300 MHz, DMSO-d₆):** δ 8.84 (s, 1H), 7.76 (s, 1H), 2.15–2.04 (m, 3H), 1.82 (d, J = 3.0 Hz, 6H), 1.71–1.53 (m, 6H). (Figure S3)

**Mass spectrometry (MS):** The compound showed very low ionization efficiency under the applied conditions (APCI, positive and negative modes), and no reliable molecular ion signal was detected.

**Infrared (IR):** A characteristic absorption band corresponding to the nitrile group was observed at 2201 cm⁻¹ (Figure S4).

**Gas chromatography (GC):** The compound eluted at 17.45 min. The observed peak exhibited tailing, likely due to limited compatibility with the stationary phase of the GC column. Nevertheless, it was the only signal detected in the region above 10 min, while earlier signals originated from the blank sample, indicating a high purity of the analyzed compound. (Figure S5, Figure S6).

**Figures:**

**
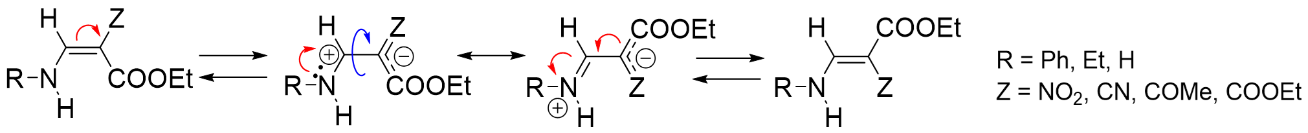
**
Figure S1. Double bond rotation between E/Z isomers by thermal isomerization mechanism.


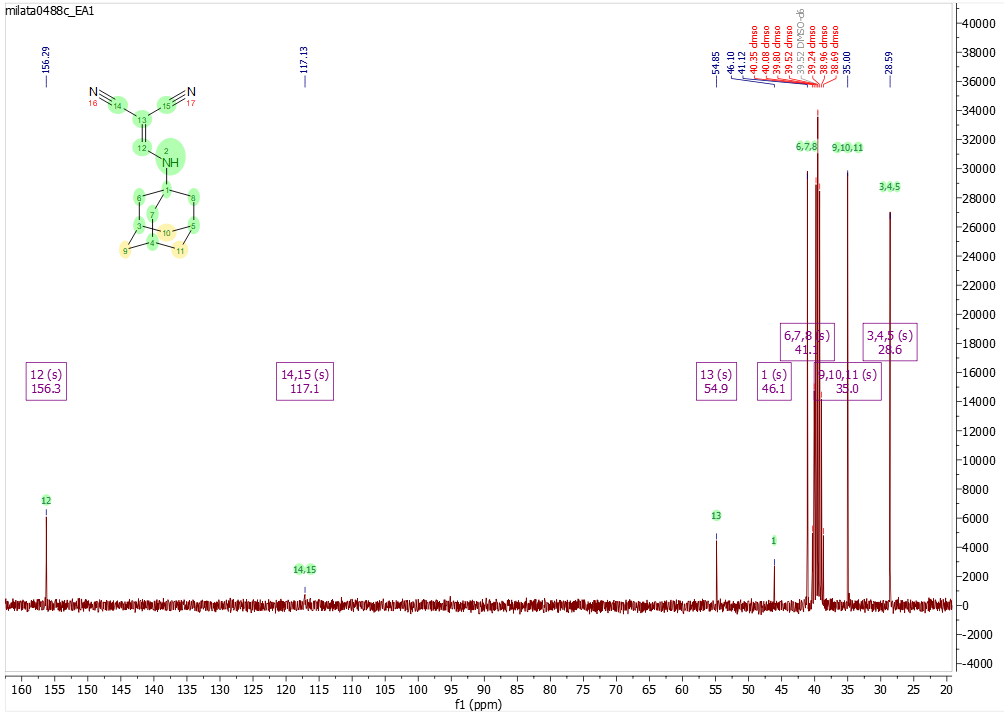

Figure S2. ¹³C NMR spectrum of E-A1.


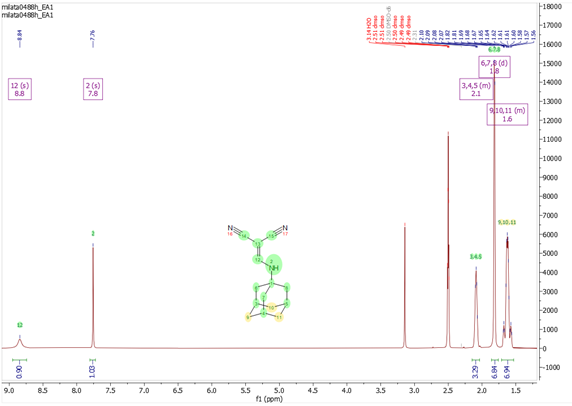
Figure S3. ¹H NMR spectrum of E-A1.


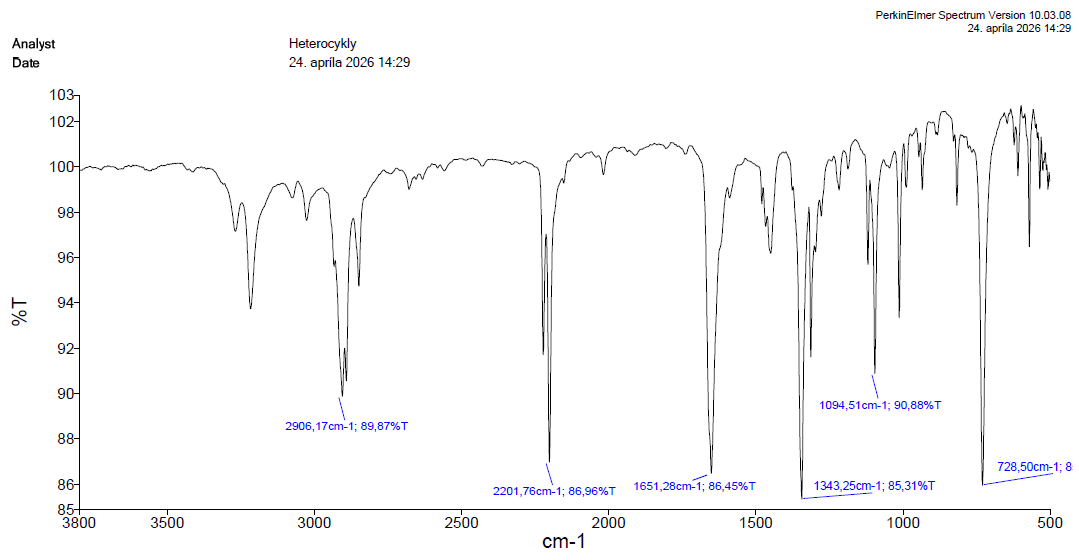

Figure S4. IR spectrum of E-A1.


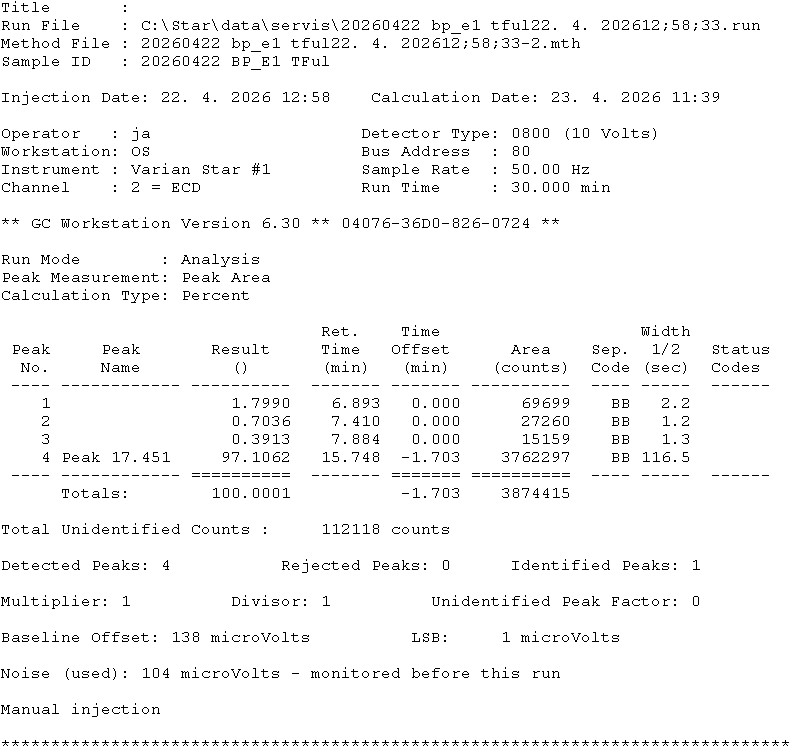

Figure S5. Report from GC for E-A1


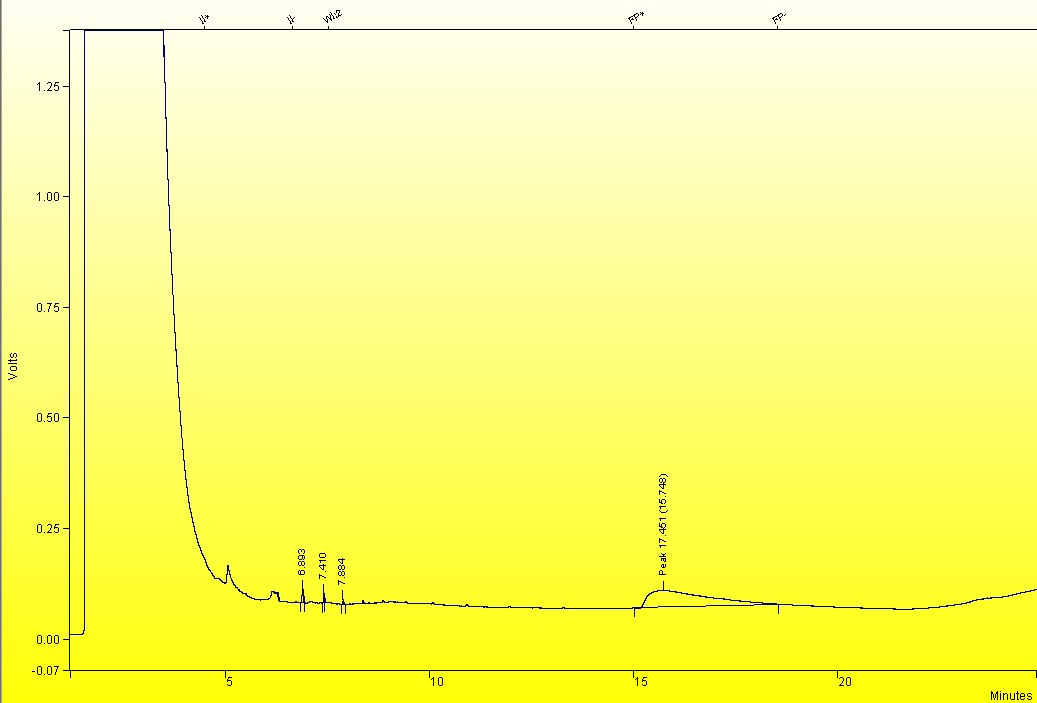


Figure S6. Full GC chromatogram for E-A1

**S3. Compound E-A2**

**Melting point:** 93–95 °C (ethyl acetate/hexanes, 1:5).

**¹H NMR (300 MHz, DMSO-d₆):** δ 9.39 (d, J = 14.9 Hz, 1H), 8.09 (d, J = 14.9 Hz, 1H), 3.61 (d, J = 14.8 Hz, 6H), 2.14–2.06 (m, 3H), 1.81 (d, J = 3.3 Hz, 6H), 1.64 (t, J = 3.2 Hz, 6H) (Figure S7).

**¹³C NMR (75 MHz, DMSO-d₆):** δ 168.62, 165.53, 154.37, 87.54, 53.20, 50.59, 50.57, 41.99, 35.14, 28.75 (Figure S8).

**Mass spectrometry (MS):** The compound exhibited a broadened LC peak, likely due to its pronounced dipole moment within a conjugated push–pull system. The recorded mass spectra correspond well with the expected fragmentation pattern. The molecular ion [M+H]⁺ was observed at m/z 294. Fragment ions at m/z 262 and 236 are consistent with the loss of methanol and methyl formate moieties, respectively. A constant offset in measured m/z values was observed across the spectrum (Figure S9-S11).

**Infrared (IR):** A characteristic absorption band corresponding to a conjugated carbonyl group (C=O) was observed at 1650 cm⁻¹ (Figure S12).

**Gas chromatography (GC):** Two signals corresponding to the compound were observed at retention times of 16.13 min and 16.83 min. Signals detected below 10 min originated from the blank sample. The relative purity of the compound, determined by GC analysis, was approximately 91% (Figure S12-15).

**Figures:**


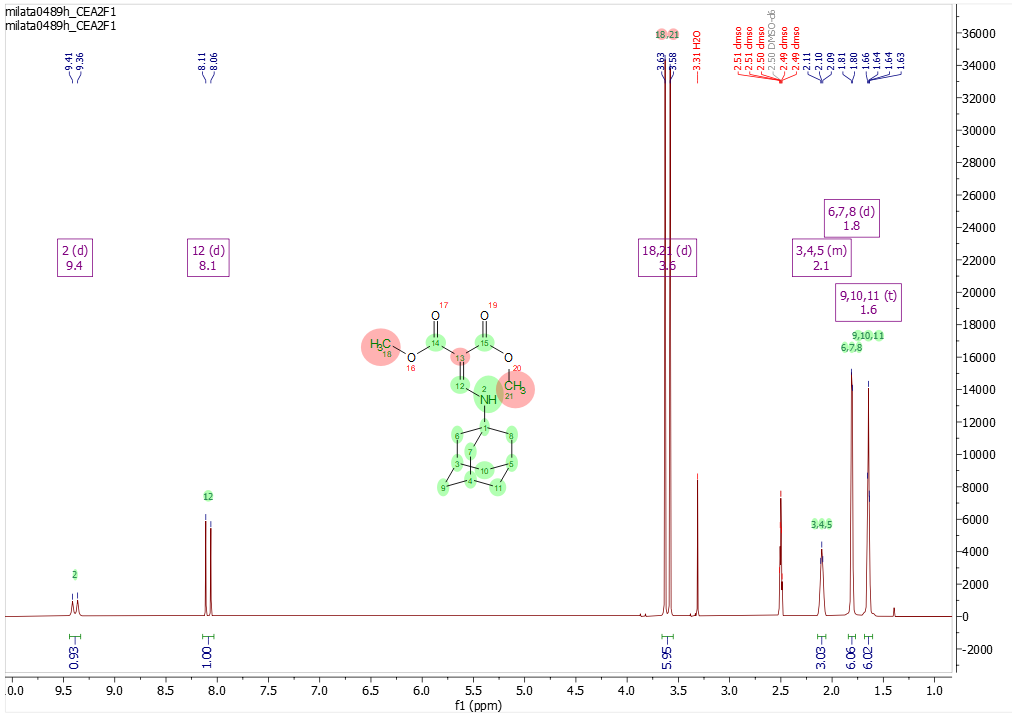


Figure S7. ¹H NMR spectrum of E-A2.


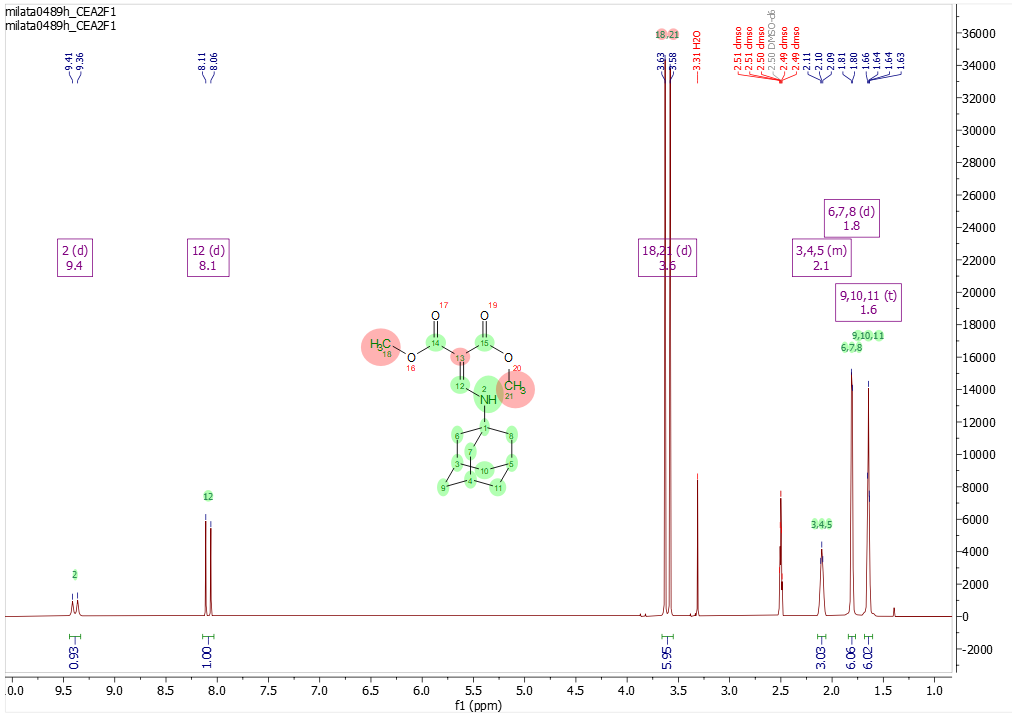

Figure S8. ¹³C NMR spectrum of E-A2.


Figure S9. Calculated m/z values.


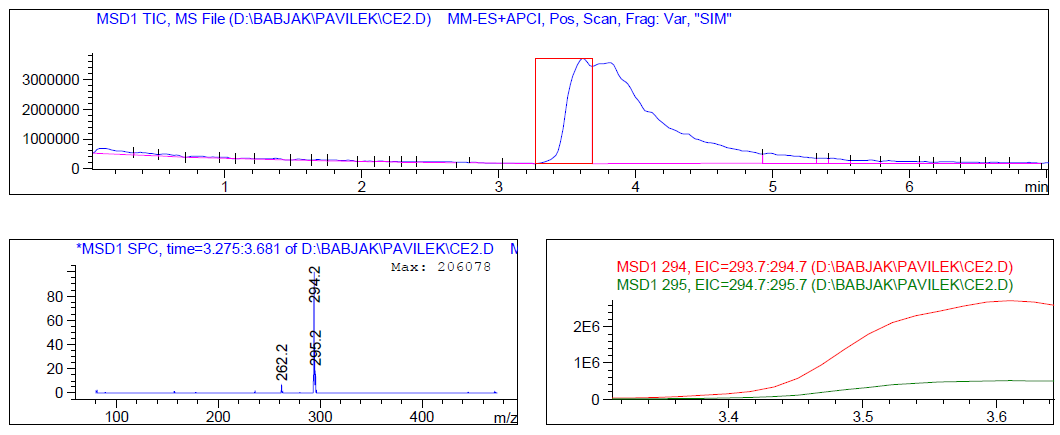

Figure S10. MS spectrum (first part of LC signal) of E-A2


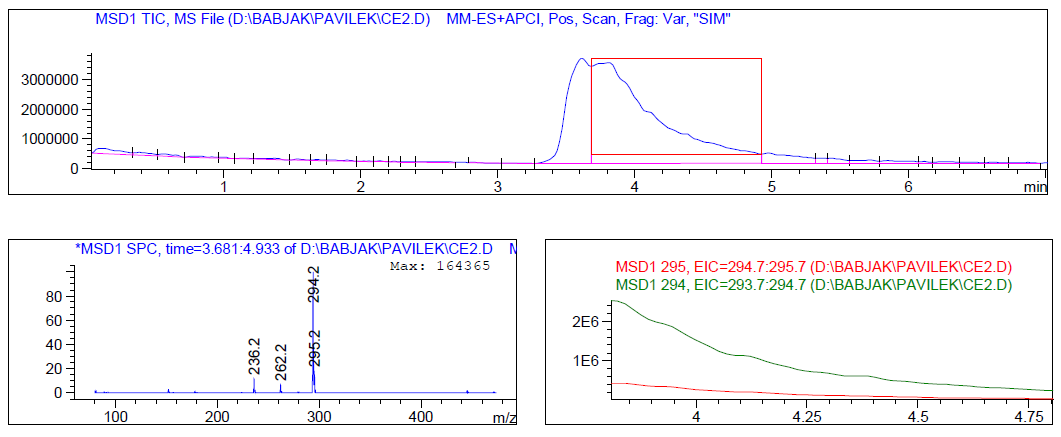
.
Figure S11. MS spectrum (second part of LC signal) of E-A2.


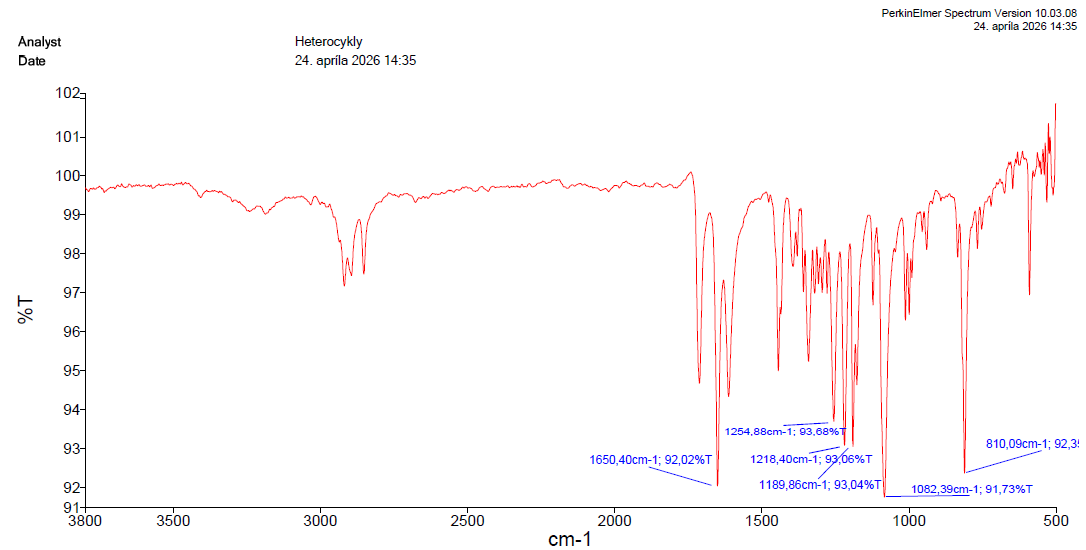

Figure S12. IR spectrum of E-A2.


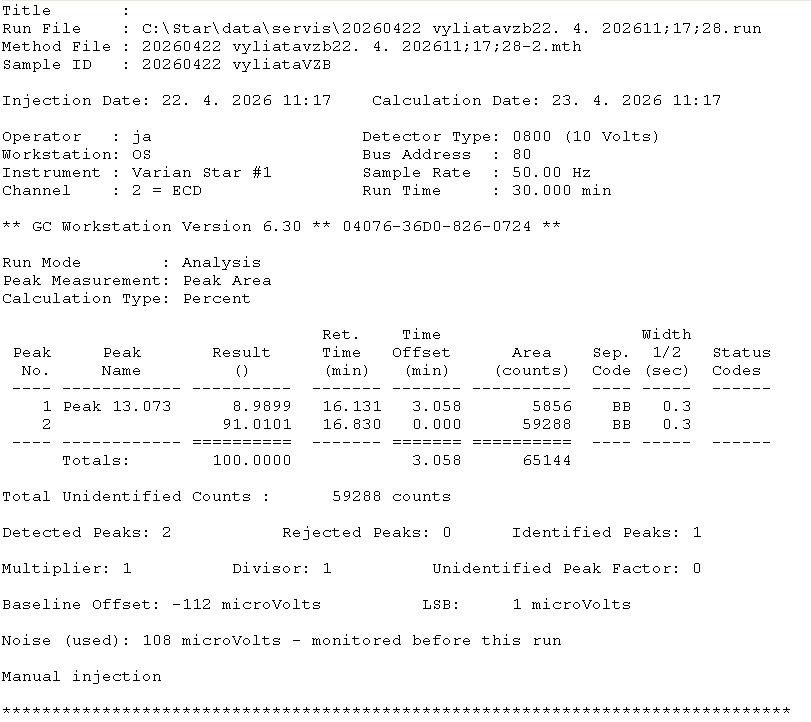

Figure S13 GC chromatogram of E-A2.


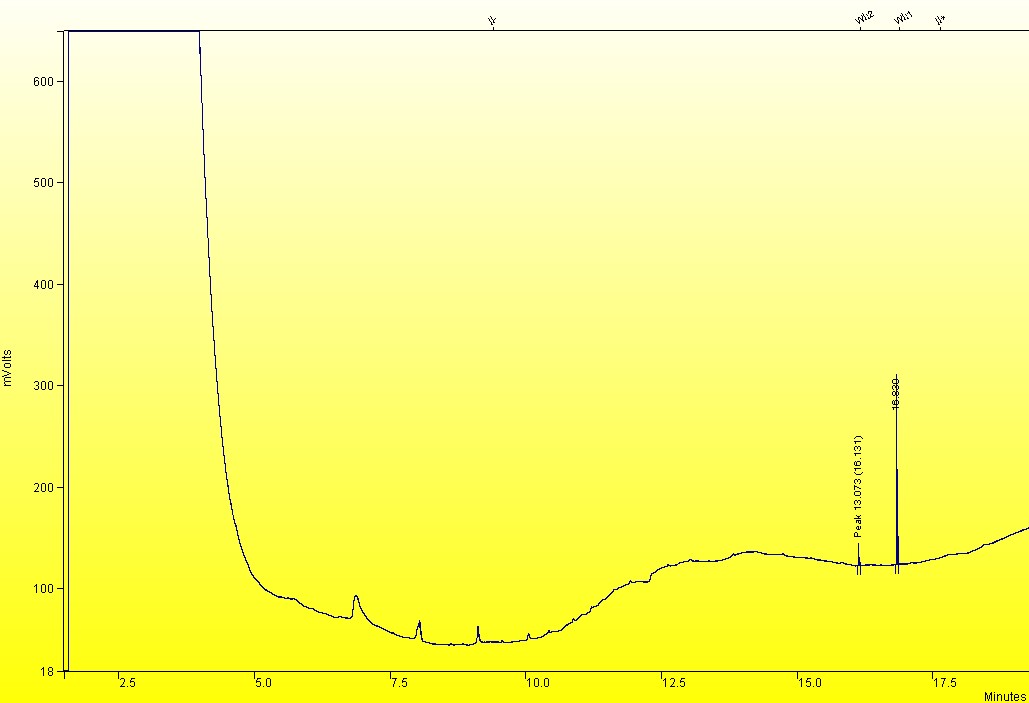

Figure 14. Full GC chromatogram for E-A2


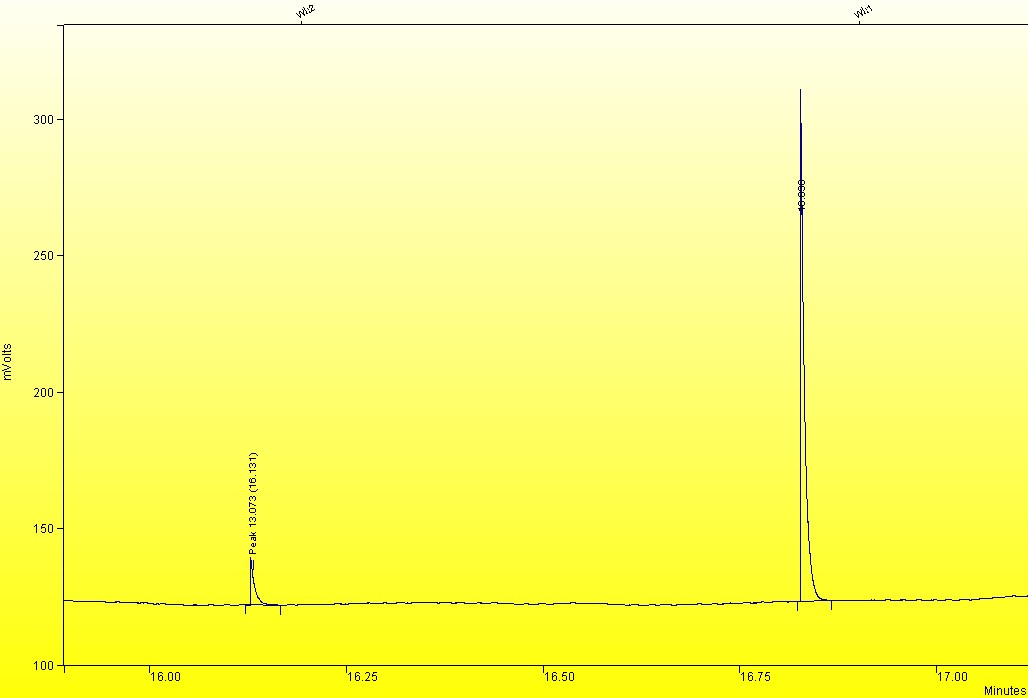


Figure 15. Zoomed GC chromatogram for E-A2

**S4. Synthesis of E-A1**

Adamantan-1-amine (0.75 g, 5 mmol) and (ethoxymethylidene)propanedinitrile (0.61 g, 5 mmol) were dissolved in ethanol (25 mL) in a 100 mL round-bottom flask. The reaction mixture was heated to reflux for 1 h. After cooling to room temperature, the product precipitated from the reaction mixture. The solid was collected by filtration and washed with a small amount of cold ethanol to afford a white powder (0.77 g, 69% yield). The compound was sufficiently pure for biological testing as confirmed by NMR and GC analysis.

*Figure S16.* Proposed reaction mechanism for the synthesis of E-A1.

**Synthesis of E-A2**

Adamantan-1-amine (0.75 g, 5 mmol) and dimethyl (methoxymethylidene)propanedioate (0.87 g, 5 mmol) were dissolved in toluene (20 mL) in a 100 mL round-bottom flask. The reaction mixture was heated to reflux for 3 h. After cooling, no precipitation was observed; however, product formation was confirmed by thin-layer chromatography (TLC) using ethyl acetate:cyclohexane (1:4) as the mobile phase.

Diatomaceous earth was added to the reaction mixture, followed by removal of the solvent under reduced pressure. The crude product was purified by column chromatography on silica gel (40 g) using a gradient of ethyl acetate:cyclohexane as eluent. The purified product was obtained as white crystalline needles (1.185 g, 82% yield).

*Figure S17*. Proposed reaction mechanism for the synthesis of E-A2.

**S5. Flow cytometry analysis (Annexin V/PI)**
Representative flow cytometry dot plots illustrating cell populations after treatment with E-A2 are shown below. Forward scatter (FSC) vs side scatter (SSC) plots were used to assess cell morphology and exclude debris, while Annexin V (FL1) vs propidium iodide (FL2) plots were used to determine viable, early apoptotic, late apoptotic, and necrotic cell populations.

Quadrant gating was applied consistently across all samples. Quadrant interpretation (Annexin V/PI staining):
LL (lower left) – viable cells (Annexin V⁻ / PI⁻);
LR (lower right) – early apoptotic cells (Annexin V⁺ / PI⁻);
UR (upper right) – late apoptotic cells (Annexin V⁺ / PI⁺);
UL (upper left) – necrotic cells (Annexin V⁻ / PI⁺).


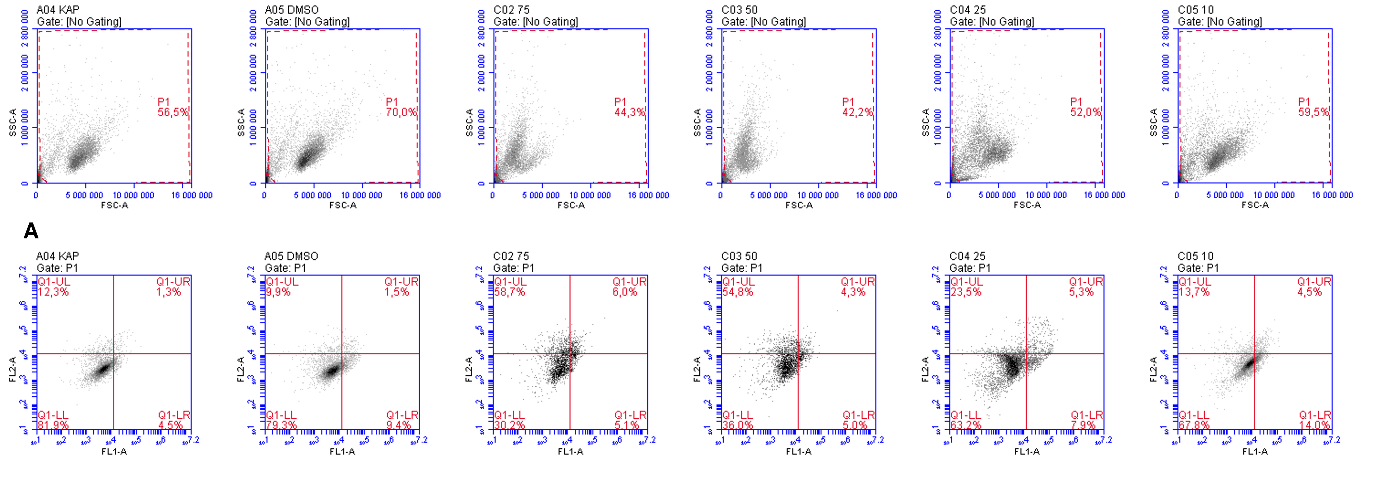

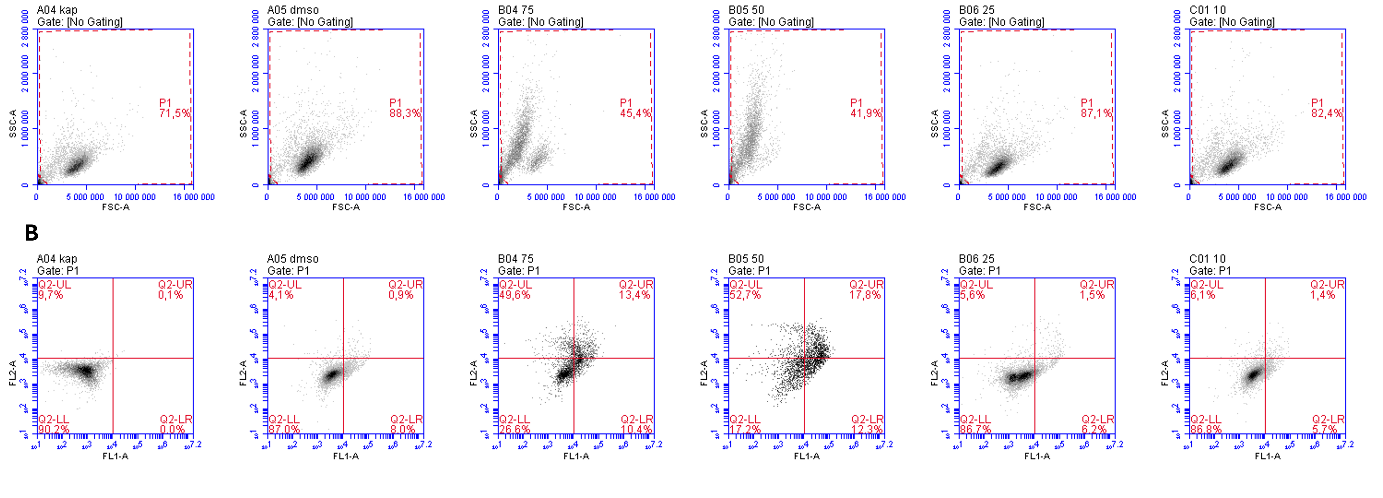


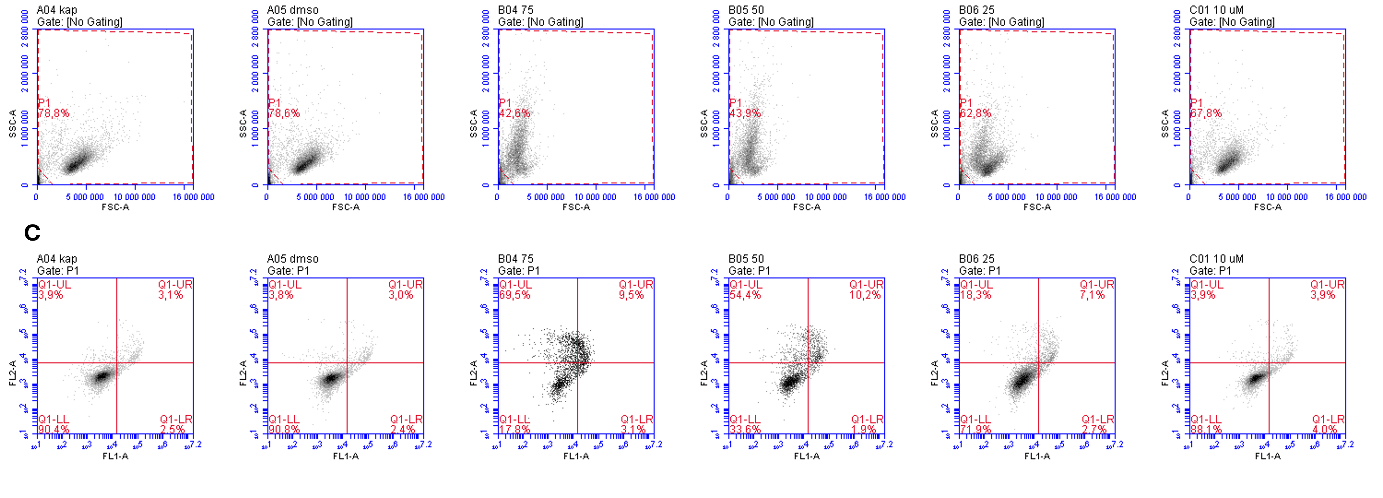

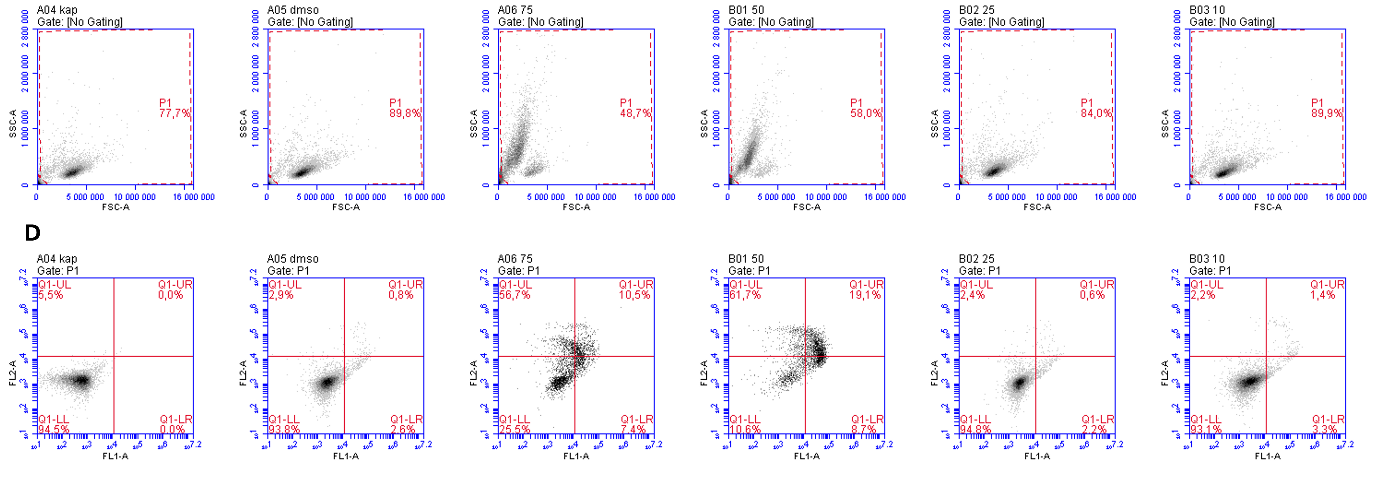


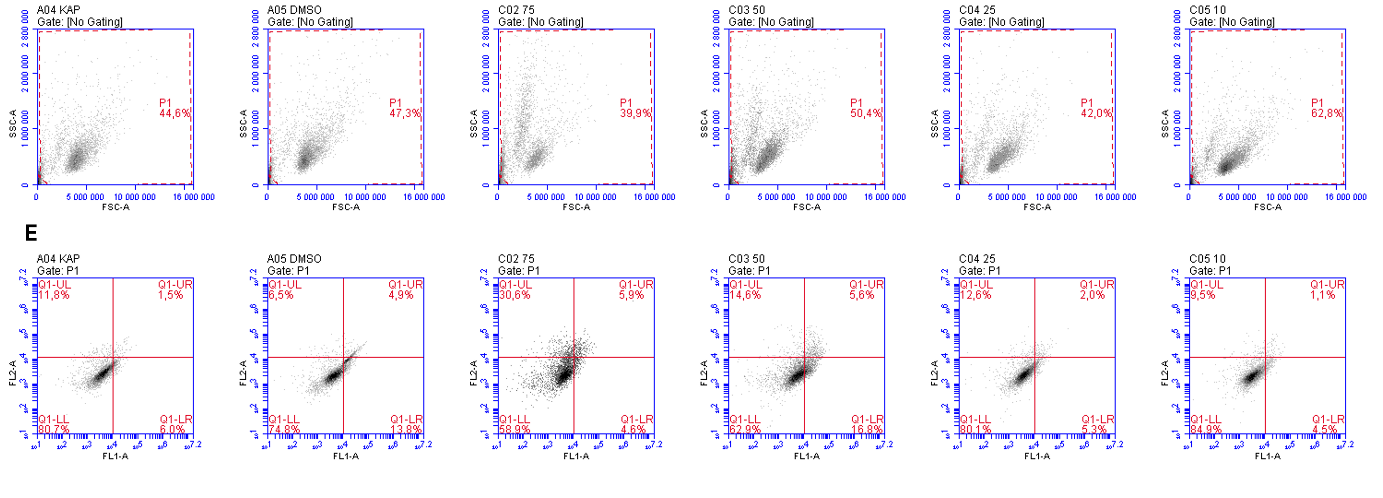

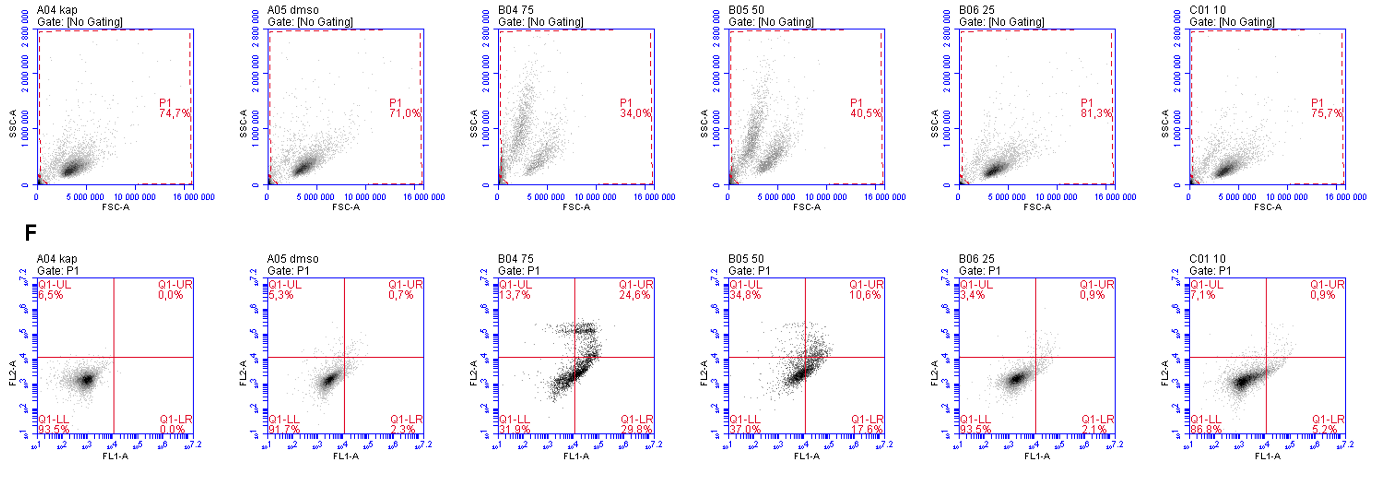


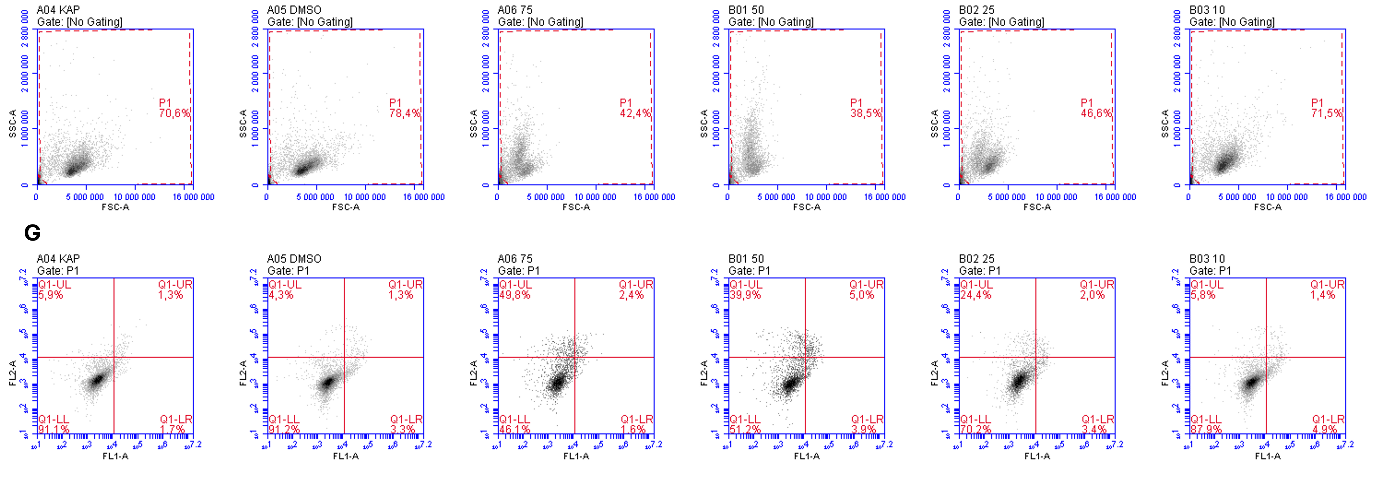

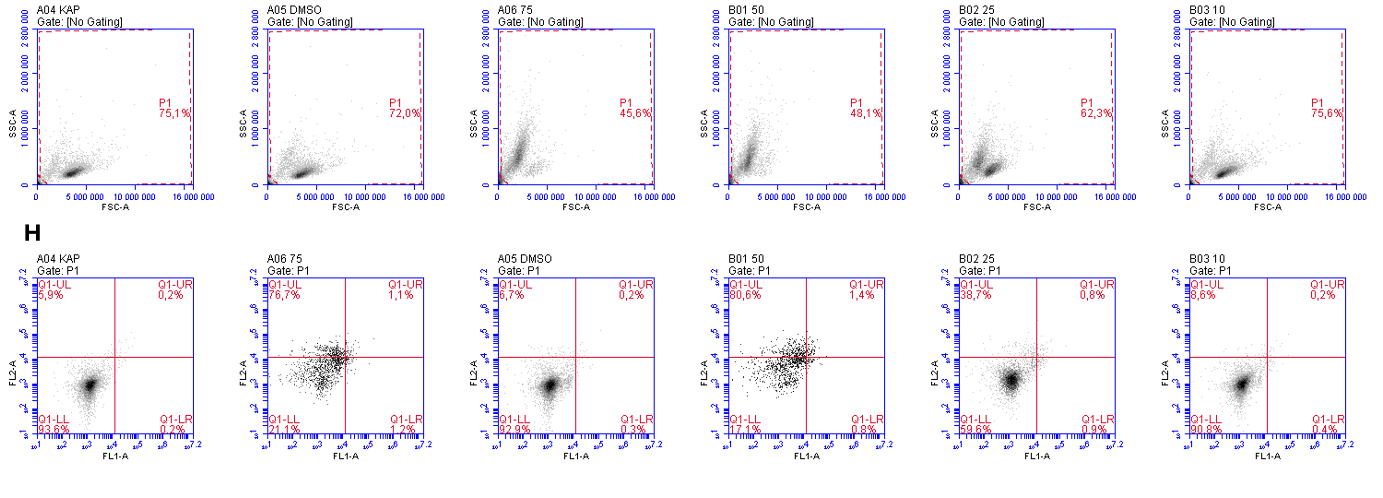


*Figure S18.* Representative FSC vs SSC (I) dot plots and Annexin V/PI (FL1 vs FL2) (II) dot plots of SKM-1 (A,B), SKM-1/VCR (C,D), MOLM-13 (E,F) and MOLM-13/VCR (G,H) cells after 24 h (A,C,E,G) and 48 h (B,D,F,H) treatment with E-A2 at indicated concentrations.


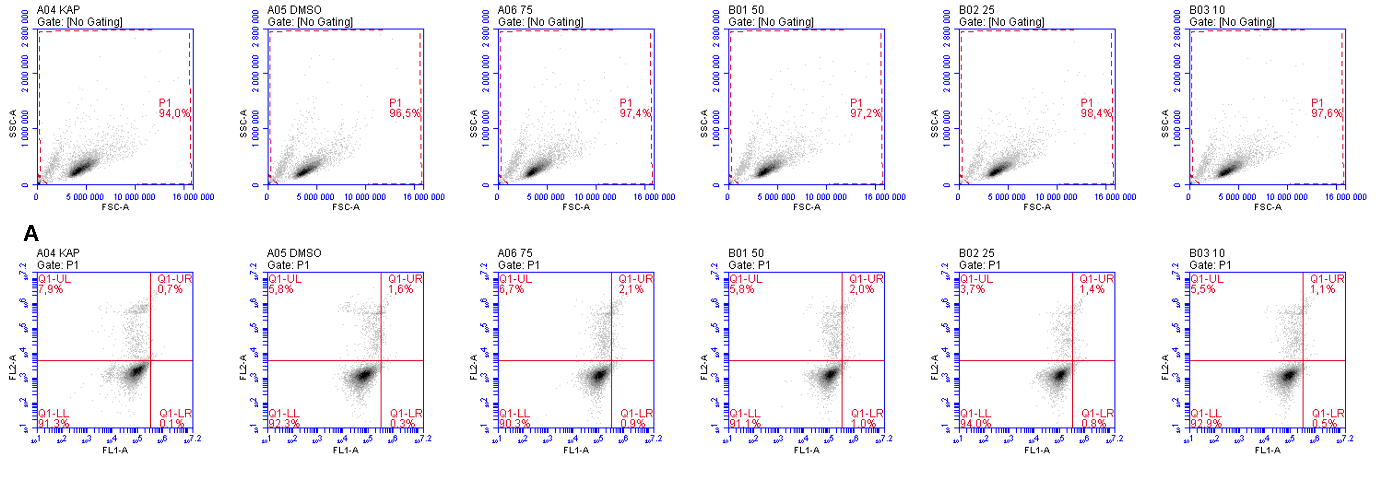
**
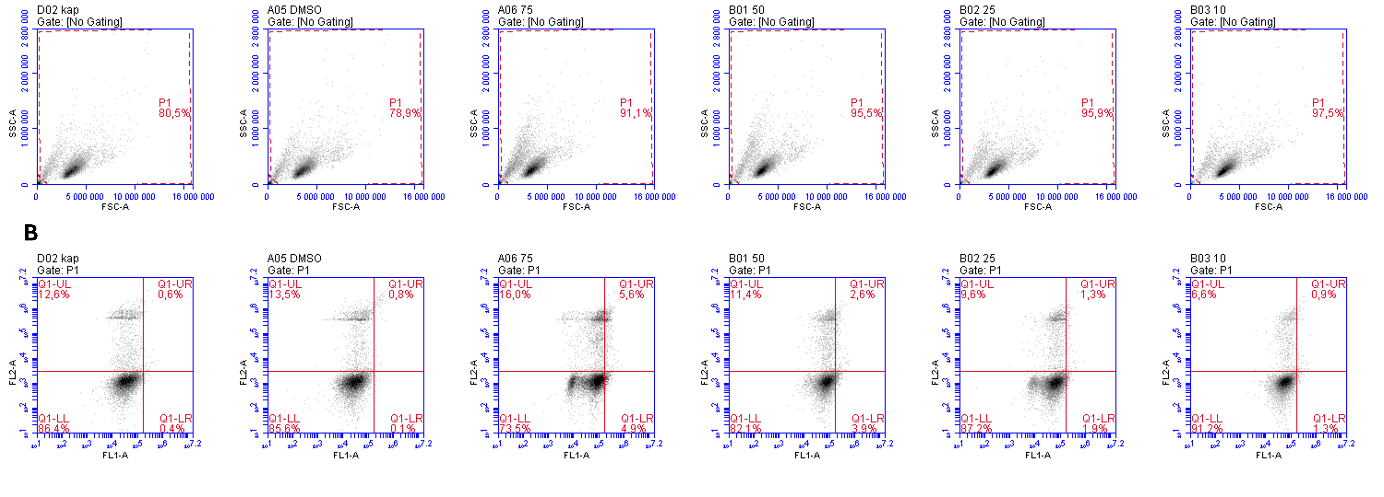
**

*
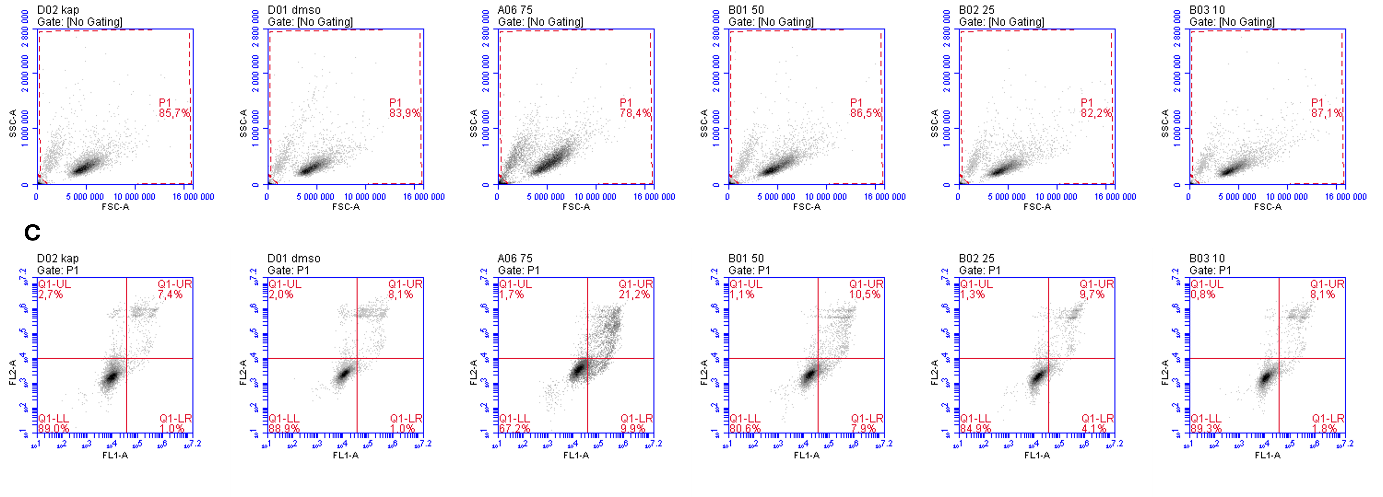

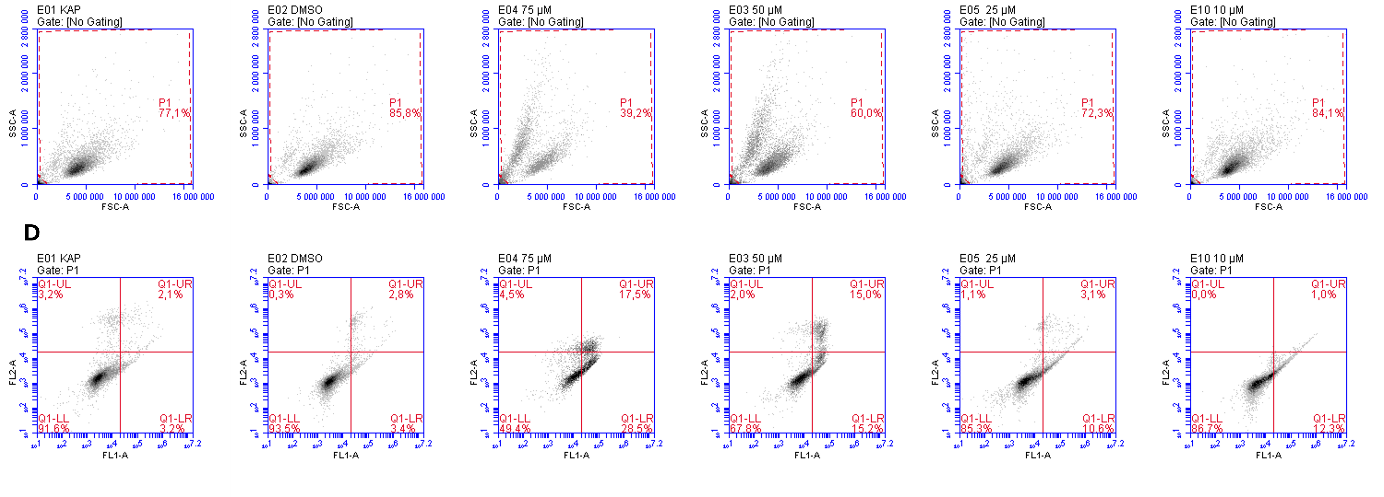
* **
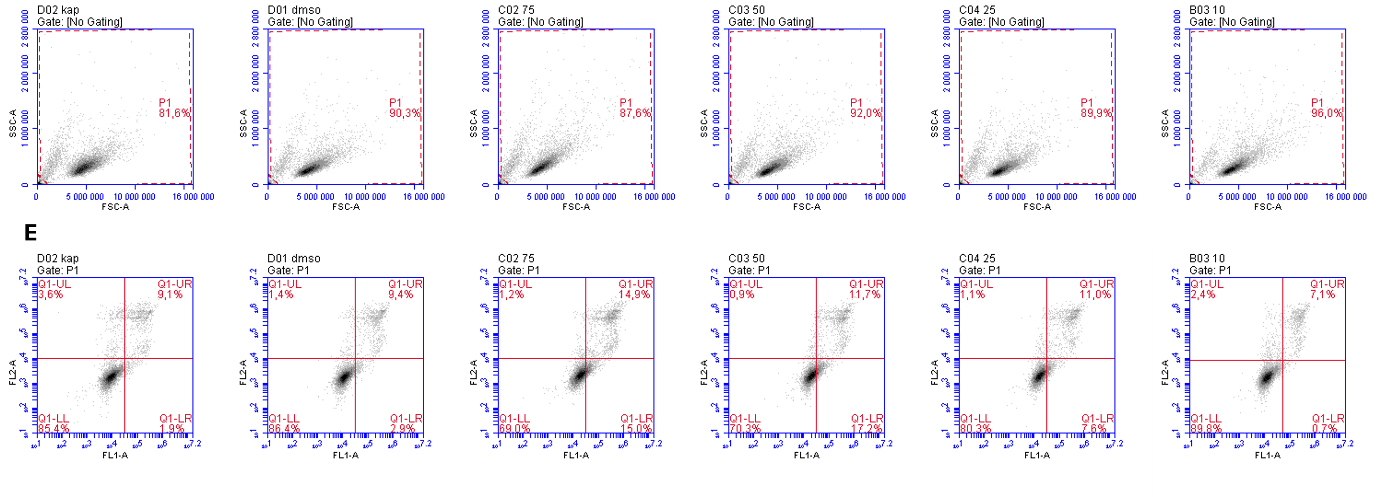
**
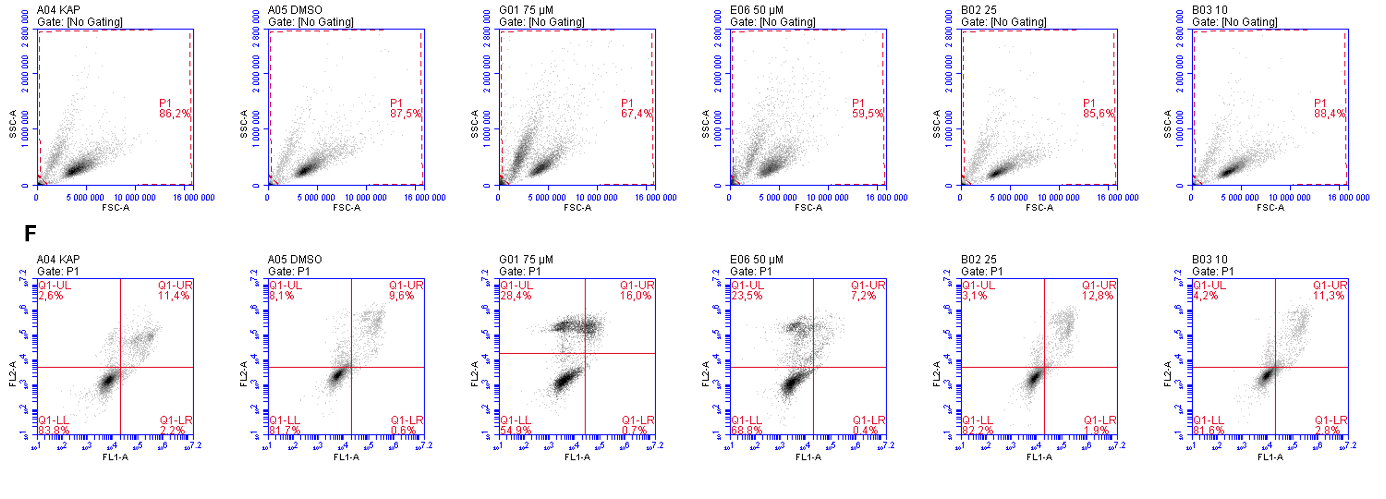


*Figure S19***.** Representative FSC vs SSC (I) dot plots and Annexin V/PI (FL1 vs FL2) (II) dot plots of L1210 S (A,B), R (C,D), and T (E,F) cells after 24 h (A,C,E) and 48 h (B,D,F) treatment with E-A2 at indicated concentrations.
